# Investigation of *Chlorella pyrenoidosa* Protein as a Source of Novel Angiotensin I-Converting Enzyme (ACE) and Dipeptidyl Peptidase-IV (DPP-IV) Inhibitory Peptides

**DOI:** 10.3390/nu13051624

**Published:** 2021-05-12

**Authors:** Yuchen Li, Gilda Aiello, Enrico Mario Alessandro Fassi, Giovanna Boschin, Martina Bartolomei, Carlotta Bollati, Gabriella Roda, Anna Arnoldi, Giovanni Grazioso, Carmen Lammi

**Affiliations:** 1 Department of Pharmaceutical Sciences, University of Milan, 20133 Milan, Italy; yuchen.li@unimi.it (Y.L.); enrico.fassi@unimi.it (E.M.A.F.); giovanna.boschin@unimi.it (G.B.); martina.bartolomei@unimi.it (M.B.); carlotta.bollati@unimi.it (C.B.); gabriella.roda@unimi.it (G.R.); anna.arnoldi@unimi.it (A.A.); giovanni.grazioso@unimi.it (G.G.); 2 Department of Human Science and Quality of Life Promotion, Telematic University San Raffaele, 00166 Rome, Italy; gilda.aiello@unimi.it

**Keywords:** ACE, bioactive peptides, *Chlorella pyrenoidosa*, DPP-IV, hypertension, microalgae

## Abstract

*Chlorella pyrenoidosa* (*C. pyrenoidosa*) is a microalgae species with a remarkably high protein content that may potentially become a source of hypotensive and hypoglycemic peptides. In this study, *C. pyrenoidosa* proteins were extracted and hydrolyzed overnight with pepsin and trypsin with final degrees of hydrolysis of 18.7% and 35.5%, respectively. By LC-MS/MS, 47 valid peptides were identified in the peptic hydrolysate (CP) and 66 in the tryptic one (CT). At the concentration of 1.0 mg/mL, CP and CT hydrolysates inhibit in vitro the angiotensin-converting enzyme (ACE) activity by 84.2 ± 0.37% and 78.6 ± 1.7%, respectively, whereas, tested at cellular level at the concentration of 5.0 mg/mL, they reduce the ACE activity by 61.5 ± 7.7% and 69.9 ± 0.8%, respectively. At the concentration of 5.0 mg/mL, they decrease in vitro the DPP-IV activity by 63.7% and 69.6% and in Caco-2 cells by 38.4% and 42.5%, respectively. Short peptides (≤10 amino acids) were selected for investigating the potential interaction with ACE and DPP-IV by using molecular modeling approaches and four peptides were predicted to block both enzymes. Finally, the stability of these peptides was investigated against gastrointestinal digestion.

## 1. Introduction

Hypertension and diabetes type 2 are two of the main risk factors for the development of cardiovascular diseases (CVD) that occur frequently together and share a substantial overlap of pathogenesis [1]. Angiotensin-converting enzyme (ACE, EC 3.4.15.1) and dipeptidyl peptidase-IV (DPP-IV, EC 3.4.14.5) are crucial enzymes involved in hypertension and diabetes, respectively. Expressed in many human tissues, ACE is associated with elevated blood pressure and is responsible for cleaving a dipeptide (HL) from the decapeptide angiotensin I to form the potent vasoconstrictor angiotensin II. Furthermore, ACE inhibits and degrades bradykinin, a potent vasodilator [2]. On the other hand, DPP-IV, a metabolic serine peptidase widely distributed in several tissues, causes the degradation and inactivation of glucagon-like peptide-1 (GLP-1) and glucose-dependent insulinotropic polypeptide (GIP), which are incretin hormones responsible for stimulating the secretion of insulin [3].

In the field of bioactive food peptides, ACE and DPP-IV inhibitory activity have been studied extensively [4,5] and many hypotensive and hypoglycemic peptides have been identified from different food matrices, such as milk, egg, ham, hempseed, lupin, and soybean, but only a few from microalgae, such as *Arthrospira platensis* (spirulina) [6,7].

Among the microalgae species available on the market, *Chlorella pyrenoidosa* stands out for its high protein content (55–60% of biomass) and balanced amino acid composition. These features indicate that this species is a sustainable food alternative as well as a potential source of bioactive peptides [8]. Several biological effects of chlorella-derived protein hydrolysates and peptides have been reported, such as antioxidant, anti-inflammatory, antihypertensive, and immunostimulant activities [9]. Although experimental and clinical studies have shown that the consumption of *C. pyrenoidosa* reduces high blood pressure, serum cholesterol, and glucose levels while also improving the immune functions [10,11,12], the peptides of this microalga are not sufficiently investigated yet.

In the light of these considerations, the first objective of this work was to obtain a total protein extract of *C. pyrenoidosa*, which was hydrolyzed using two common gastrointestinal enzymes, i.e., pepsin and trypsin, in order to produce a peptic (CP) and a tryptic (CT) hydrolysate. The composition of each hydrolysate was assessed by HPLC-ESI-MS/MS, using a peptidomic approach, and the potential hypotensive and hypoglycemic activities were evaluated, initially measuring the ability of both hydrolysates to inhibit in vitro the ACE and DPP-IV activities and, afterwards, by assessing their effects on the modulation of the same enzymes expressed on Caco-2 cells. The positive results obtained in these experiments suggested the presence of some very active multifunctional peptides within both hydrolysates. In order to identify the active species, docking and molecular dynamics (MD) studies were performed to simulate the interaction between the peptides and the protein targets, and to hierarchize the most promising ones. Finally, the hydrolysates were submitted to simulated gastrointestinal digestion (GI) in order to verify whether the candidate peptides were sufficiently stable in digestion.

## 2. Materials and Methods

### 2.1. Reagents

All chemicals and reagents were of analytical grade and from commercial sources. Detailed information is shown in Supplementary Materials.

### 2.2. Microalgae Biomass

*C. pyrenoidosa* dry powder was purchased from Qingdao Lang Yatai Company Limited (Qingdao, China). The manufacturer declares that they had cultivated it in photoautotrophic conditions in outdoor runway pools and that the dry powder had been prepared by spray drying. According to the product information, the dry powder of *C. pyrenoidosa* mainly consist of 57.3% of crud protein, 14.5% of fat, 6.2% of carbohydrate, 5.7% of ash, 10.0% of crude fiber. It is noticed that microalgae easily accumulate heavy metals, which could bind to the internal binding sites of some proteins and peptides. Caution needs to be undertaken to ensure they are free from heavy metal. For this reason, in our study, we have used food-grade microalgae produced following good manufacturing practices which guarantee that the level of heavy metals do not exceed the requirement of standards.

### 2.3. Ultrasound and Heating Assisted Protein Extraction

*C. pyrenoidosa* powder was defatted overnight with hexane (ratio 1:20 *w/v*) under magnetic stirring. After drying, the defatted powder (0.5 g) was suspended in 10 mL of lysis buffer (8 M urea, 1% CHAPS, 20 mM DTT in 0.1 M of NH4HCO3). The mixture was placed in ice and treated with an ultrasonic cell disruptor for 6 min (5 s at 50 W, 23 kHz frequency pulses followed by 5 s of cool-down period). Then the sonicated suspension was heated for 15 min and cleared via centrifugation at 7200× *g* (4 °C, 30 min). The supernatant was collected and dialyzed against ddH_2_O at 4 °C for 48 h and then stored at −20 °C until analysis. The protein concentration was detected by Bradford assay. By SDS-PAGE, the chlorella protein extract was profiled as previously reported [13].

### 2.4. C. pyrenoidosa Protein Hydrolysis and Peptide Sequencing by LC-ESI MS/MS

The enzymatic hydrolysis of *C. pyrenoidosa* proteins was performed using trypsin and pepsin. For trypsin and pepsin digestion, the pH of the protein extracts was adjusted to pH 8 and 2, respectively, by adding 1 M NaOH or 1 M HCl. The trypsin and pepsin solutions were added to the protein extracts at a 1:50 (*w/w*) E/S ratio. After overnight digestion (16 h), trypsin was inactivated by heating at 95 °C for 5 min, whereas the peptic digestion was blocked by adjusting the pH to 8. Each hydrolysate was passed through ultrafiltration membranes with a 3 kDa cut-off, using a Millipore UF system (Millipore, Bedford, MA, USA). Recovered peptides were lyophilized and stored at −80 °C until use. The degree of hydrolysis (DH) of each hydrolysate was measured by the o-phthaldialdehyde (OPA) assay [13]. CT and CP hydrolysates were analyzed by HPLC-CHIP-ESI-MS/MS as previously reported [14]. For the peptide identification, the MS/MS data were analyzed by a Spectrum Mill Proteomics Workbench (Rev B.04.00, Agilent), consulting the *C. vulgaris* database (310 entries) downloaded from the UniProtKB-SwissProt. The use of this database instead of *C. pyrenoidosa* is justified by the phylogenetic proximity of these microalgae. Pepsin and trypsin were selected as cutting enzymes, respectively. Two missed cleavages were allowed to each enzyme used; peptide mass tolerance was set to 1 Da and fragment mass tolerance to 0.8 Da. Auto-validation strategy on both peptide and protein polishing modes was performed using FDR cut-off ≤1.2%.

### 2.5. Biological Evaluation of the Peptic and Tryptic Hydrolysates

#### 2.5.1. In Vitro Measurement of the ACE Inhibitory Activity

In order to assess the ACE-inhibitory activity, the peptic and tryptic hydrolysates were tested by using HHL as a mimic substrate for angiotensin 1 and the produced hippuric acid (HA) was analyzed by HPLC, as previously reported [15]. The detailed procedures are shown in the Supplementary Materials.

#### 2.5.2. In Vitro Measurement of the DPP-IV Inhibitory Activity

The in vitro experiments were carried out in a half-volume 96-well solid plate (white) using conditions previously optimized [16]. Further details are provided in Supplementary Materials.

#### 2.5.3. Cell Cultures

Caco-2 cells, obtained from INSERM (Paris, France), were routinely sub-cultured following conditions which are detailed in the Supplementary Materials.

#### 2.5.4. Cellular Measurement of the ACE Inhibitory Activity

A total of 5 × 10^4^ Caco-2 cells/well were seeded in 96-well plates for 24 h. In the following day, cells were treated with 100 μL of hydrolysates from chlorella proteins digested by pepsin (CP) and trypsin (CT) (in the concentration range from 1.0 to 5.0 mg/mL) or vehicle in growth medium for 24 h at 37 °C. Afterwards, the ACE1 Activity Assay Kit (Biovision, Milpitas Blvd., Milpitas, CA, USA) was used to evaluate the ACE inhibitory activity. The procedure is described in Supplementary Materials.

#### 2.5.5. Cellular Measurement of DPP-IV Inhibitory Activity

Caco-2 cells (5 × 10^4^/well) were seeded in black 96-well plates with clear bottoms and cultured for 24 h. Afterwards, spent media was removed and CP and CT hydrolysates (1.0, 2.5, and 5.0 mg/mL), sitagliptin at 1.0 μM (positive control), or vehicle in growth medium were separately used to treat Caco-2 cells for 24 h at 37 °C. Treatment media were then removed and cells were washed with 100 μL of PBS without Ca^++^ and Mg^++^, and 100 μL of Gly-Pro-AMC substrate (Cayman Chemical, Ann Arbor, MI, USA) at the concentration of 50.0 μM in PBS without Ca^++^ and Mg^++^ were added in each well. Fluorescence signals (ex./em. 350/450 nm) were detected using the Synergy H1 fluorescent plate reader from Biotek every 1 min for 10 min.

### 2.6. Docking and MD Simulations on the Inhibitory Peptides in Complex with ACE and DPP-IV

The molecular modeling studies were aimed to predict the binding modes of CT and CP peptides on human ACE and DPP-IV enzymes. These studies relied on the models’ preparation stages, the docking studies, the MD simulations, and the binding free energy estimation as detailed below.

#### 2.6.1. Computational Models Setup

Before performing docking calculations, the OPLS3e force field was assigned to ACE, DPP-IV, and the peptides by means of the “Protein Preparation Wizard” and LigPrep tools available in Maestro software (release 2020-2, Schrodinger Inc.: New York, NY, USA). The coordinates of the human ACE and DPP-IV enzymes were retrieved from the Protein Data Bank (PDB, http://www.rcsb.org accessed on 11 April 2021). In particular, the X-ray crystallographic structures of the human-testicular ACE in complex with captopril (PDB ID: 1UZF) and the human DPP-IV, in complex with Diprotin A(IPI) (PDB ID: 1NU8), were selected for our studies [17,18]. The protein preparation phase involved the following steps: (1) pre-processing of the imported protein structures applying the default parameters, as recommended for the minimal processing; (2) modifying the structure by removing all the water molecules and undesired ligands (i.e., the ones different from captopril or IPI); (3) H-bond optimizations and assignment of the residue protonation states at the pH of 7.0 (Epik tools) [19]; (4) finally, performing the restrained minimization of the protein heavy atoms to a root mean square deviation (RMSD) of 0.3 Å, calculated considering as reference the starting model geometry. To prepare the ligands, Captopril and Diprotin A (IPI) were extracted from the structures retrieved from PDB. For the short peptides, the 3D builder tool was utilized to build the extended conformation of the eleven selected peptides, starting from their primary structures. For all ligands, the LigPrep tool was used to produce the structure endowed with the lowest potential energy value for docking analysis. The ionization and tautomeric states at the pH of 7.0 ± 2.0 were generated by means of Epik tool. The original chirality (*S*) of the peptides was retained to produce data in line with the experimental data.

#### 2.6.2. Docking Calculations of the Selected Peptides on ACE and DPP-IV

The GLIDE algorithm was employed to perform the docking calculations [20]. First, the “receptor” grids were generated in the pre-processed protein structure of ACE and DPP-IV, respectively. Next, docking studies were performed on both catalytic sites of the enzymes (the ones identified by the presence of captopril and IPI in the X-ray structures) applying the default parameters, while the Standard Precision (SP) algorithm was used to score the attained docking poses. The ligand/enzyme complexes attained by docking were further optimized by the “Perform post-docking minimization” option in which the bond lengths, bond angles, as well as the torsion angles of the attained complexes were optimized to simulate the ligand–receptor “induced fit” process. Finally, five docking poses per peptide were visually inspected and the one with the lowest docking score was furtherly considered for MD simulations.

#### 2.6.3. MD Simulations and Trajectory Analysis

All solvated enzyme/peptide complexes needed to perform MD simulations were prepared by building an orthorhombic TIP3P box of water molecules [21], by means of the proper tool implemented in Maestro. The complex models were then submitted to 200 ns-long MD simulations at the temperature of 300 K, pressure of 1.01325 bar (NPT conditions), and the Desmond algorithm was utilized to perform these calculations [22]. A trajectory for each complex was recorded during the MD simulations time, wherein the snapshots of complex were extracted at every 1 ns, producing 200 snapshots totally. The MD trajectories were visually inspected by the VMD program [23] and, after analyzing the peptide backbone RMSD changes on each MD trajectory, Molecular Mechanics/ Generalized Born Surface Area (MM/GBSA) were performed to calculate the binding free energy of each peptide [24]. These calculations were performed on the trajectory frames in which the peptide under investigation displayed the lowest conformational mobility, estimated by plotting the RMSD values of the Calpha atoms over the simulation time.

### 2.7. Evaluation of Peptide Stability toward Simulated Gastrointestinal Digestion

After having shifted the pH to 2.0, the CP and CT solutions were added with pepsin (4 mg/mL in NaCl using E/S ratio 1:100) and incubated at 37 °C for 60 min under continuous shaking. Then, the pH was adjusted to 8.0 with 1 M NaOH and pancreatin (4 mg/mL in ddH_2_O) was added (E/S ratio 1:100) for performing the digestion at 37 °C for 15 min. The enzymatic reaction was blocked by heating at 95 °C for 10 min and the enzymes were completely removed by centrifugal ultrafiltration (3 kDa cutoff). This process was repeated without adding pepsin and pancreatin in order to prepare the controls. The stability of the four screened peptides was assessed through multiple reaction monitoring (MRM) mass spectrometry. The detailed information of the peptide analysis is available in Supplementary Materials.

### 2.8. Statistical Analysis

Statistical analyses were performed by Student’s t-test, Mann–Whitney test, and one-way ANOVA (GraphPad Prism 9, GraphPad Software, La Jolla, CA, USA) followed by Dunnett’s and/or Tukey’s post-hoc test. More details are provided in Supplementary Materials.

## 3. Results

### 3.1. Optimization of the Protein Extraction and Hydrolysis

**Figure 1:**
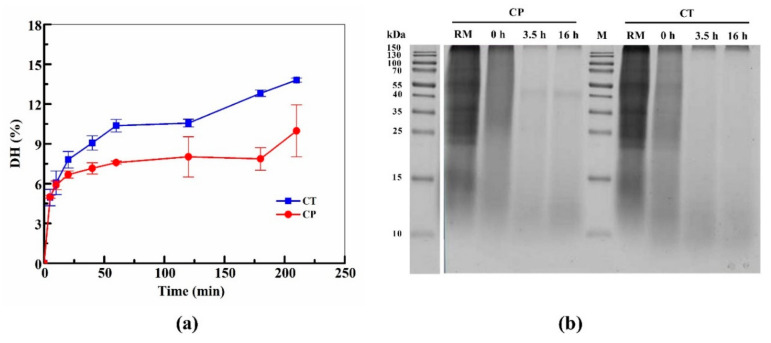
Degree of the hydrolysis (DH) trend and digestion efficiency of protein from *C. pyrenoidosa*. (**a**) DH at different time points within the first 3.5 h of enzymatic digestion. (**b**) SDS-PAGE analysis of hydrolysates sampled at different hydrolysis time points. RM: raw protein extract; CP: *C. pyrenoidosa* proteins digested by pepsin; CT: *C. pyrenoidosa* protein digested by trypsin.

The cell walls of green microalgae are known to have rigid wall components as the main protective barrier against the environment [25]. In order to efficiently break the cells and to improve the protein extraction from the *C. pyrenoidosa* matrix, a protocol with specific conditions was optimized (the data is shown in Supplementary Materials). At the end of this investigation, a highly concentrated urea solution (8 M) buffer coupled with ultrasonication and heating achieved the best protein yield. Ultrasound-assisted extraction has good physical effects to disrupt the cell wall by the process of cavitation, while heating at reasonable temperature can increase mass transfer and the solubility of protein [26,27]. With this optimized method, the concentration of total protein extract from *C. pyrenoidosa* was 18.34 mg/mL. For the hydrolysis, two enzymes, i.e., trypsin and pepsin, were employed, whose effectiveness was monitored by detecting the DH (%). Figure 1a shows the trend of the DH during the first 3.5 h, indicating that in both cases the maximum rate of hydrolysis was achieved in the first 30 min. After overnight digestion (16 h), the final DH of the CP and CT reached 18.7% and 31.8%, respectively. Correspondingly, SDS-PAGE was used to compare the protein profile of raw and enzymatically digested samples for both the CP and CT group. In Figure 1b, the RM lane shows the protein composition of raw total protein extracts without the addition of enzymes. The most intense protein bands were detected in the range between 25–170 kDa. During hydrolysis, both these intense protein bands had almost disappeared after the first 3.5 h.

### 3.2. Peptide Profile by LC-MS/MS

The peptide profiles of CP and CT were characterized by nano-ESI-MS/MS. In total, 47 peptides were identified in the CP sample and 66 in the CT sample (Table S1). Since several studies have demonstrated that significant biological effects are exerted by low molecular weight peptides, short peptides (aa ≤ 10) were selected as a targeted dataset for further investigations [28]. Table 1 lists the identified peptide sequences with the number of amino acids (aa) ≤ 10, among which 5 were from the CP sample and 6 from the CT sample. All the peptides were numbered as Pep1 to Pep11. Among these short peptides, 6 peptides (Pep1, Pep4, Pep6, Pep7, and Pep11) were abundant, since they showed high spectrum intensity (>10^8^), accounting for the main percentage of the protein hydrolysates. Hydrophobicity of each selected peptide was calculated by the Wimley–White scale. Five peptides (Pep2, Pep3, Pep7, Pep9, and Pep10) showed relatively high hydrophobicity, with values equal to +12.19, +18.27, +10.37, +26.37, and +19.00 Kcal/mol, respectively.

### 3.3. In Vitro and Cellular ACE Inhibitory Activity

**Figure 2:**
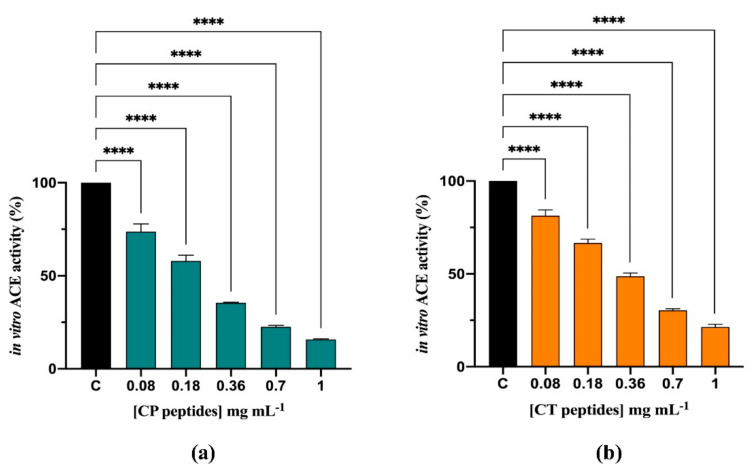
Evaluation of the in vitro inhibitory effects of CP (**a**) and CT (**b**) hydrolysates on ACE. Bars represent the sd of 3 independent experiments in duplicate. **** *p* < 0.0001 versus Control sample (C).

**Figure 3:**
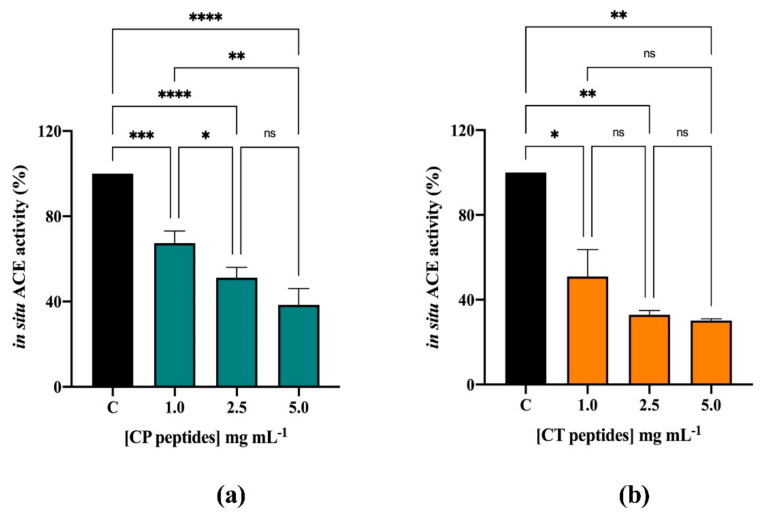
Evaluation of the inhibitory effects of CP (**a**) and CT (**b**) hydrolysates on ACE expressed by Caco-2 cell membranes. Bars represent the SD of 3 independent experiments in duplicate. **** *p* < 0.0001, *** *p* < 0.001, ** *p* < 0.01, * *p* < 0.05, ns, no significantly different versus Control sample (C).

The inhibitory effects of CP and CT hydrolysates (0.08–1.0 mg/mL) on the in vitro ACE activity were evaluated using the porcine recombinant form of the enzyme. Figure 2a,b shows that both CP and CT hydrolysates efficiently inhibited the ACE activity by 84.2 ± 0.37% and 78.6 ± 1.7%, respectively, at 1.0 mg/mL. The CP hydrolysate was more active, having a better IC_50_ than CT (0.21 mg/mL vs. 0.32 mg/mL). In order to assess the effects of the CP and CT hydrolysate at cellular level, human intestinal Caco-2 cells were treated with both hydrolysates (at 1.0–5.0 mg/mL) for 24 h. By performing MTT experiments in this range of concentration, any cytotoxic effect has been observed (Figure S1). After cell lysis, the ACE activity was measured in the presence of a fluorescent substrate. In this assay, both hydrolysates inhibited the cellular ACE activity with a dose-response trend. In more detail, CP hydrolysate reduced the enzyme activity by 32.6 ± 5.6%, 48.9 ± 4.9, and 61.5 ± 7.7%, respectively, at 1.0, 2.5, and 5.0 mg/mL (Figure 3a), whereas CT hydrolysate reduced it by 49 ± 12.7%, 67.1 ± 2.0%, and 69.9 ± 0.8%, respectively, at the same concentrations (Figure 3b).

### 3.4. In Vitro and Cellular DPP-IV Inhibitory Activity

**Figure 4:**
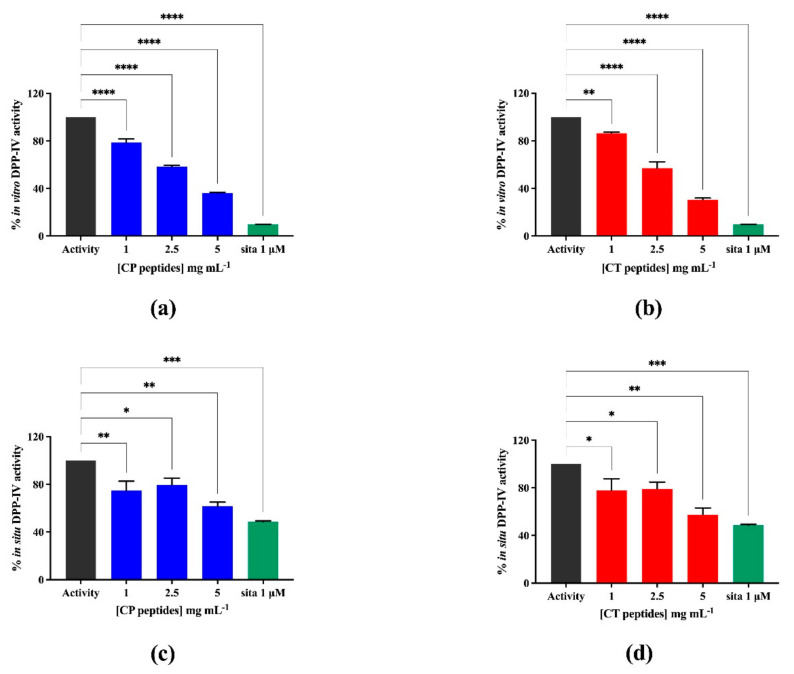
Evaluation of the inhibitory effects of CP and CT hydrolysates on DPP-IV. (**a**,**b**) in vitro activity of human recombinant DPP-IV, (**c**,**d**) cellular activity of DPP-IV expressed on Caco-2 cell membranes. Bars represent the average ± SD of 3 independent experiments in duplicates. **** *p* < 0.0001, *** *p* < 0.001, ** *p* < 0.01, * *p* < 0.05 versus untreated sample (Activity). sita: sitagliptin, positive control, at 1 μM.

For evaluating the DPP-IV inhibitory activity of the hydrolysates, in vitro experiments were preliminarily performed by using the purified recombinant DPP-IV enzyme and H-Gly-Pro-AMC as a substrate. The reaction was monitored by measuring the fluorescence signals (465 nm) deriving from the release of a free AMC group after the cleavage of H-Gly-Pro-AMC catalyzed by DPP-IV. Figure 4a,b indicates that both CP and CT hydrolysates significantly inhibited the DPP-IV activity in vitro: CP reduced the DPP-IV activity by 21.3 ± 2.9, 41.5 ± 0.9, and 63.7 ± 0.5%, respectively, at 1.0, 2.5, and 5.0 mg/mL, whereas CT by 13.7 ± 0.8, 43.1 ± 5.4, and 69.6 ± 1.4%, respectively, at the same concentrations. Then, the DPP-IV inhibitory activity was assessed in cellular experiments, using Caco-2 cells that are an improved tool for the screening of DPP-IV inhibitors, since they are a reliable model of intestinal epithelial cells and express high levels of DPP-IV [29,30]. The same concentrations applied in the in vitro assay were used here, with the awareness that, by performing MTT experiments in this range of concentration, any cytotoxic effect can be observed (Figure S1). By monitoring the same fluorescent reaction, clear DPP-IV inhibitory effects were observed as shown by Figure 4c,d. The CP hydrolysate dropped the cellular DPP-IV activity by 25.3 ± 7.9, 20.5 ± 5.7, and 38.4 ± 3.4% at 1.0, 2.5, and 5.0 mg/mL, respectively, and CT by 22.3 ± 9.8, 20.5 ± 5.7, and 42.5 ± 5.7% at 1.0, 2.5, and 5 mg/mL, respectively. These results indicate that both hydrolysates are less active at the cellular level than in vitro.

### 3.5. In Silico Studies

After having identified by LC-MS/MS the most abundant short peptides, it was decided to predict their molecular interactions with ACE and DPP-IV enzymes by molecular modeling studies. The Maestro software was used in this aim, since it is a validated platform allowing the easy building of the simulation systems and performance of docking calculations, MD simulations, and trajectory post-processing analysis.

Initially, the accuracy of the docking protocol was ascertained by performing docking calculations on the ligands found in the X-ray structures, i.e., captopril and Diprotin A (IPI) in complex with ACE and DPP-IV, respectively [31]. As expected, the GLIDE protocol implemented in Maestro displayed excellent results; in fact, the RMSD value of the docked pose of captopril was 0.7640 Å, calculated comparing the X-ray binding mode (Figure S2A–C, Supplementary Materials). On the other hand, acceptable results were also attained for IPI in DPP-IV, for which a slightly higher RMSD value of 2.7390 Å was attained comparing the best scored docking solution with the X-ray conformation of IPI (Figure S2D–F, Supplementary Materials). However, since typically docking predictions follow in a RMSD range of 2–3 Å, we considered the performance of the GLIDE docking protocol to be satisfactory; it was then applied to predict the binding modes of the short peptides attained by *C. pyrenoidosa* hydrolysis. By using GLIDE, the complexes composed by each peptide and ACE or DPP-IV were consequently obtained. For each complex, the docking poses with the lowest value of the glide score (higher predicted affinity) were selected for further MD simulations and binding free energy calculations. Table 2 summarizes the calculated binding free energy values and the docking scores of the peptide/ACE and peptide/DPP-IV complexes. For each enzyme, the peptides were ranked by the value of the binding free energy value, because these values were obtained from the dynamic interaction of the ligands and the enzymes, providing more detailed information in terms of the complex stability (see the Materials and Methods Section). Generally, the more negative the binding free energy value, the better stability and binding affinity between the docked peptides and enzyme were predicted, indicating the highest potential ACE or DPP-IV inhibiting effects [32]. Hence, according to the data on Table 2, four peptides (Pep2, Pep 7, Pep8, and Pep10) displayed the lowest binding free energy value on both ACE and DPP-IV enzymes, indicating that these peptides could potentially display multifunctional activity, since they could exert inhibitory effects on both enzymes, involved in different pathologies.

Figure S3 (Supplementary Materials) represents the 2D views of the predicted interactions of the most promising peptides (Pep2, Pep 7, Pep8, and Pep10) in complex with ACE or DPP-IV. As it is shown in Figure S3a–d, the docking results indicate that the four peptides were docked into the catalytic site via multiple interactions, some of which are critical in the active sites of ACE, since some known ACE inhibitors are bound to the enzyme, making several contacts with the same residues. For example, captopril binds to Gln281, His353, Lys511, His513, Tyr520, whereas lisinopril binds to His353, Ala354, Glu384, Lys511, His513, Tyr520, and Tyr523. This enhances the possibility that the four docked peptides inhibit ACE activity by competitively blocking the catalytic domain of ACE structure [17,33], as suggested by these in silico studies. By our simulations, in fact, His353, Ala354, and Tyr523 were bound to Pep2 (FLKPLGSGK), Glu162, Glu384, His353, His513, and Glu411 were bound to Pep7 (QIYTMGK), His353 and Tyr523 were involved in the interaction with Pep8 (FLFVAEAIYK), and Glu162, Gln281, Ala354, and His387 interact with Pep10 (QHAGTKAK). Moreover, Pep2, Pep7, and Pep10 was docked in an area within 3.5 Å from the zinc ion. In particular, Pep7 created a salt bridge with the zinc ion and this interaction contributed −2.3 kJ/mol (Glide metal) to the overall estimated glide score. On the other hand, Figure S3e–h (Supplementary Materials) shows the docking complexes between each peptide and DPP-IV, revealing the key interactions. In particular, we have found that Pep2 could be bound to Glu205, Glu206, Tyr547, and Trp629, Pep7 to Glu205, Glu206, Arg125, Ser630, Tyr662, and Tyr547, Pep8 to Arg125, Tyr666, and Trp629, and Pep10 to Ser209, Trp629, Arg125, and Tyr547. Glu205 and Glu206, reported as an important double Glu motif, generated a H-bond/salt bridge network with residue Arg125, essentially being responsible for the substrate recognition and selectivity. Other critical residues in the binding site, i.e., Tyr547, Trp629, Ser630, Tyr662, and Tyr666, play essential roles in the catalytic mechanism of DPP-IV [3,34] and, as shown by docking calculations, are involved in the binding of our peptides. On this basis, it was possible to hypothesize that these peptides probably act by blocking the substrate recognition and the catalytic function of DPP-IV, acting with a biological mechanism similar to that of Diprotin A (IPI) and some DPP-IV inhibitors, such as linagliptin and sitxagliptin [35,36].

### 3.6. Evaluation of the Stability of the Peptides towards Simulated Gastro-Intestinal (GI) Digestion

**Figure 5:**
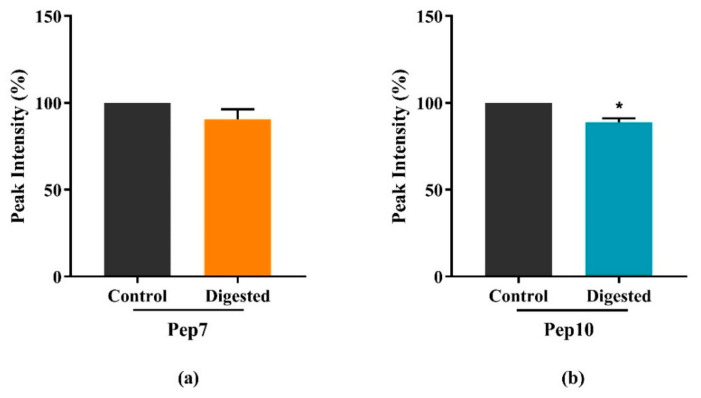
Stability evaluation of Pep7 (**a**) and Pep10 (**b**) stability by in vitro simulated GI digestion. Bars represent the average ± SD of 3 independent experiments in duplicates. Data were analyzed by Student’s t-test and the Mann–Whitney test. * *p* < 0.05 versus undigested samples (control).

To evaluate the stability of the target peptides, the CP and CT hydrolysates were submitted to simulated GI digestion with pepsin and pancreatin and the resulting solutions were analyzed by a targeted MRM assay, in comparison with the undigested samples. Whereas all four peptides were detected in the undigested samples (control) as shown by their MS/MS fragmentation spectra shown in Figure S3, their behaviors in the digested samples were diverging. In fact, Pep7 and Pep10 were easily identified in the digested samples, whereas no traces of Pep2 and Pep8 could be detected. Figure 5 indicates that, after the digestion with pepsin and pancreatin, the intensities of Pep7 and P10 were decreased by 9.5 ± 5.8% and 11.1 ± 2.1%, respectively, compared to the undigested control group.

## 4. Discussion

Using a multidisciplinary strategy, *C. pyrenoidosa* protein was investigated as a source of peptides active against ACE and DPP-IV targets, in line with the preclinical and clinical evidences suggesting that the consumption of *C. pyrenoidosa* may be useful for the prevention of cardiovascular disease.

Both CP and CT hydrolysates drop in vitro the ACE activity with a dose-response trend, although CP is slightly more active than CT. Both hydrolysates display also a comparable inhibitory activity at cellular level, but less efficiently than in vitro indicating that they are affected by the peptidases expressed on the membrane of intestinal cells.

Owing to the complex composition of these protein hydrolysates, it may be presumed that they contain peptides with different biological activities [37]. In fact, the activity of a protein hydrolysate depends on its total composition, including active and inactive species, and on possible synergistic or antagonist effects. This may explain why these hydrolysates are active on two different targets, such as ACE and DPP-IV. The reduced activity in the cellular assays is probably explained by the degradation induced by Caco-2 cells [38]. In fact, the intestinal brush border is a very complex physiological environment where many active proteases and peptidases are expressed that might metabolize food peptides modulating their bioactivity. Therefore, this organ acts not only as a major physiological barrier against the external environment, permitting the absorption of valuable nutrients, but also actively participates in the modulation of the physico-chemical profiles of food protein hydrolysates, through the metabolic activity of its proteases.

Similar results have previously been obtained on peptic (SP) and tryptic (ST) hydrolysates of spirulina protein. SP and ST hydrolysates inhibit in vitro the ACE activity with IC_50_ of 0.1 and 0.28 mg/mL, and on Caco-2 cells with IC_50_ of 2.7 and 2.8 mg/mL, respectively. In addition, SP reduces the DPP-IV activity by 64.6% in vitro and 31.7% on Caco-2 cells, while ST reduces it by 74.2% in vitro and 39.8% on Caco-2 cells (at the dose of 5 mg/mL) [14]. In addition, the tryptic digest of phycobiliprotein purified from spirulina inhibits the DPP-IV activity by 95.8% in vitro, whereas it inhibits it by 44% in situ at 5 mg/mL [39].

It is also possible to compare these results with those obtained testing hempseed and soybean protein hydrolysates produced with the same enzymes (pepsin and trypsin) and in the same conditions. In more detail, at 1.0 mg/mL, the tryptic (HT) and peptic (HP) hydrolysates from hempseed proteins inhibit in vitro the DPPIV activity by 17.5 ± 2.7% and 32.0 ± 6.2%, respectively, and at cellular level by 15.5 ± 1.8% and by 22.5 ± 0.19%, respectively [40]. A peptic soybean hydrolysate (SoP) reduces in vitro the DPP-IV activity by 16.3 ± 3.0% and 31.4 ± 0.12%, respectively, at 1.0 and 2.5 mg/mL, whereas a tryptic one (SoT) by 15.3 ± 11.0% and 11.0 ± 0.30%, respectively at 1.0 and 2.5 mg/mL. The SoP hydrolysate is 2 times less active on Caco-2 cells (37% inhibition at 5.0 mg/mL) than in vitro on the DPP-IV enzyme (31.4% inhibition at 2.5 mg/mL).

The favorable activity observed suggested the possibility of developing an in silico strategy to identify at least some potential bioactive peptides. Among commonly used in silico methods, molecular docking permits researchers to evaluate the interaction between peptides and complex proteins, in order to predict the binding free energy values and the putative binding modes [41]. A similar work-flow has been applied for discovering novel ACE inhibitory peptides from stone fish protein hydrolysates [42]. Besides the molecular docking study, here also MD simulations were performed in order to evaluate the stability of the complex during a specified simulation time, improving greatly the accuracy of the predictions.

In this way, four peptides (Pep2, Pep7, Pep8, and Pep10) were finally selected as the best candidates for the biological activity, since they form stable complexes with ACE and DPP-IV. Their substructures, composed by three or more amino acid residues, were searched by in the SciFinder database (https://scifinder.cas.org/, accessed on 10 May 2021). Pep2 (FLKPLGSGK) contains two reported ACE-inhibitory motifs, i.e., PLG and LKP. The former, isolated from Alaskan pollack skin, is an ACE inhibitory peptide with an in vitro IC_50_ value equal to 4.74 mM [43], whereas the latter, released from dried bonito as well as chicken, was reported to be an ACE inhibitor with a good in vitro activity (IC_50_ 0.32 μM), which also effectively decreases the systolic blood pressure in spontaneously hypertensive rats (SHR) [44,45]. Within Pep8 (FLFVAEAIYK), the fragment AIYK has been identified in a peptic hydrolysate from wakame protein, and shown to reduce the ACE activity in vitro with an IC_50_ of 213 μM and to induce a significant decrease of blood pressure in SHR [46]. Interestingly, some the AIYK fragments, i.e., IYK, YK, IY, are also in vitro ACE inhibitors, with IC_50_ values of 177, 610, and 2.65 μM, respectively [46]. Using the BIOPEP database (http://www.uwm.edu.pl/biochemia/index.php/pl/biopep, accessed on 10 May 2021), it was confirmed that Pep2 and Pep8 contain specific motifs within their own sequences that account for the ACE inhibitory activity. Contextually, the same peptides contain also some DPP-IV inhibitory fragments; i.e., KP, FL, and PL for Pep2 and VA, FL, AE, and YK for Pep8.

In addition, the potential activities of the selected peptides may be supported by their structural features. In fact, to be a potent ACE/DPP-IV inhibitor, a short peptide with 2–10 amino acids is preferred. In addition, the presence of numerous hydrophobic amino acid residues is thought to be important, since the hydrophobic properties enhance the interaction between peptides and the functional hydrophobic pocket in ACE and DPP-IV enzymes. In line with this principle, Pep2, Pep7, and Pep10 have relatively high calculated hydrophobicity values (Table 1). Moreover, peptides with an aromatic residue (Trp, Tyr, Pro, and Phe), positive charged residue (His, Arg, and Lys), or branched side chain (Leu, Ile, and Val) as their C-terminal amino acids are very likely to be good ACE inhibitors. Indeed, all selected peptides have a positive charged Lys residue as their C-terminal amino acid. Instead, to be potent DPP-IV inhibitors, the peptides should respect the following rules: (1) contain a Pro residue (at the first, second, third, or fourth N-terminal position), flanked by Leu, Val, Phe, Ala, and Gly (a rule followed by Pep2, FLKPLGCGK); (2) have a hydrophobic or aromatic amino acid at N-terminus (as in Pep2, FLKPLGCGK, and Pep8, FLFVAEAIYK).

Since peptides are potentially susceptible to hydrolysis by the brush border peptidases expressed on the apical membranes of intestinal epithelium, as already reported in the literature [38,47], the stability of Pep2, Pep7, Pep8, and Pep10 towards gastric digestion was here evaluated. After peptic followed by the pancreatic hydrolysis, our results revealed that Pep7 and Pep10 showed good GI tolerance whereas Pep2 and Pep8 were unstable and degraded during digestion, indicating that Pep7 and Pep10 are more likely to reach the biological targets exercising their potential bioactivities after oral administration.

It is important to underline that in domains which may be responsible in either of the ACE and DPP-IV inhibitory activities, it is highly probable that these are multifunctional peptides [37]. The evaluation of the gastrointestinal stability has shown that Pep7 and Pep10 are probably the most interesting species for possible future exploitation. Finally, it is useful to underline that this is the first study that has investigated the potential of using *C. pyrenoidosa* protein to generate antihypertensive and hypoglycemic peptides targeting ACE and DPP-IV activities.

## Figures and Tables

**Table 1:** Short peptides (≤10 aa) identified from CP and CT by LC-MS/MS.

	Peptides	Spectrum Intensity	m/z (Charge)	MW (Da)	Hydrophobicity (Kcal/mol) ^a^	Protein Precursor
	*From CP*					
Pep1	LLGRC	5.09E + 08	281.04 (2)	561.318	+8.34	Broad-range acid phosphatase DET1
Pep2	FLKPLGSGK	2.75E + 07	473.99 (2)	946.573	+12.19	Serine/threonine-protein kinase
Pep3	MSANHDAGGS	1.24E + 07	473.63 (2)	946.369	+18.27	Uncharacterized protein
Pep4	LLSKT	5.31E + 08	281.64 (2)	561.361	+8.91	GIY-YIG catalytic domain-containing endonuclease
Pep5	LLTKS	2.72E + 07	280.94 (2)	561.361	+8.91	Uncharacterized protein
	*From CT*					
Pep6	ILGCR	2.01E + 09	280.96 (2)	561.318	+8.47	Uncharacterized protein
Pep7	QIYTMGK	7.32E + 08	280.96 (3)	840.429	+10.37	Uncharacterized protein (Fragment)
Pep8	FLFVAEAIYK	2.78E + 07	601.28 (2)	1200.667	+8.37	Ribulose bisphosphate carboxylase large chain
Pep9	EAERGGDGR	2.00E + 07	474.11 (2)	946.434	+26.37	Uncharacterized protein
Pep10	QHAGTKAK	2.68E + 07	280.93 (3)	840.469	+19.00	Phosphatidylserine synthase 2
Pep11	LLSTK	5.17E + 08	281.11 (2)	561.361	+8.91	Uncharacterized protein

**^a^** Hydrophobicity was calculated by PepDraw tool (http://www.tulane.edu/~biochem/WW/PepDraw/, accessed on 10 May 2021), according to the method of the Wimley–White scale, 1996.

**Table 2:** Binding free energy values and docking score of the best poses obtained by the application of the computational protocol on the Chlorella-derived peptides interacting with (**a**) ACE and (**b**) DPP-IV targets.

(a) ACE as the Receptor					(b) DPP-IV as the Receptor				
Peptide			Binding Free Energy (kJ/mol)	Docking Score			Peptide	Binding Free Energy (kJ/mol)	Docking Score
*CT*	Pep8	FLFVAEAIYK	−101.6	−7.415	*CT*	Pep10	QHAGTKAK	−74.1	−2.918
*CT*	Pep7	QIYTMGK	−83.3	−10.807	*CT*	Pep7	QIYTMGK	−64.4	−7.503
*CP*	Pep2	FLKPLGSGK	−81.	−11.458	*CP*	Pep2	FLKPLGSGK	−60.2	−8.099
*CP*	Pep3	MSANHDAGGS	−78.8	−8.997	*CT*	Pep8	FLFVAEAIYK	−59.9	−8.415
*CT*	Pep11	LLSTK	−53.9	−10.601	*CP*	Pep1	LLGRC	−56.1	−7.61
*CT*	Pep10	QHAGTKAK	−51.9	−6.411	*CP*	Pep5	LLTKS	−53.4	−6.566
*CP*	Pep5	LLTKS	−49.5	−9.605	*CP*	Pep4	LLSKT	−47.7	−8.139
*CT*	Pep6	ILGCR	−47.9	−9.179	*CT*	Pep9	EAERGGDGR	−46.8	−6.777
*CT*	Pep9	EAERGGDGR	−46.4	−7.712	*CT*	Pep11	LLSTK	−45.4	−7.565
*CP*	Pep1	LLGRC	−32.7	−8.297	*CP*	Pep3	MSANHDAGGS	−43.3	−9.345
*CP*	Pep4	LLSKT	−9.2	−9.354	*CT*	Pep6	ILGCR	−35.1	−4.632

## Data Availability

The data used to support the findings of this study are available from the corresponding author upon request.

## Supplementary Materials

The following are available online at https://www.mdpi.com/article/10.3390/nu13051624/s1, Table S1: LC-ESI-MS/MS based identification of (a) tryptic and (b) peptic peptides from *C. pyrenoidosa* protein hydrolysis. Figure S1: Effects of CP and CT on HepG2 cell viability; Figure S2: Figure validation of the molecular docking by comparing the docking pose and crystal structure of ACE-captopril and DPP-IV-IPI complexes, respectively. Figure S3: Predicted binding modes of short peptides within ACE and DPP-IV. Figure S4: Electrospray ionization–tandem mass spectrometry spectra of [M+H] ^+^ ions of (a) Pep2, (b) Pep 7, (c) Pep8, and (d) Pep10, respectively.
