# Supplementary material for: Investigation of Chlorella pyrenoidosa Protein as a Source of Novel Angiotensin I-Converting Enzyme (ACE) and Dipeptidyl Peptidase-IV (DPP-IV) Inhibitory Peptides
Source: Nutrients. 2021 May 12;13(5):1624. doi: 10.3390/nu13051624 (PMC8151766; doi:10.3390/nu13051624)
Supplement: Supplementary file 1 [file nutrients-13-01624-s001.zip › nutrients-1200622-supplementary.pdf]

Investigation of *Chlorella pyrenoidosa* protein as a sources of novel angiotensin I-converting enzyme (ACE) and dipeptidyl peptidase- IV (DPP-IV) inhibitory peptides

Yuchen Li[1], Gilda Aiello[2], Patrizia Locatelli[3], Enrico Mario Alessandro Fassi[1], Giovanna Boschin[1], Carlotta Bollati[1], Martina Bartolomei[1], Gabriella Roda[1], Anna Arnoldi[1], Giovanni Grazioso[1], Carmen Lammi[1*]

[1] Department of Pharmaceutical Sciences, University of Milan, 20133 Milan, Italy
[2] Department of Human Science and Quality of Life Promotion, Telematic University San Raffaele, Rome, Italy
[3] Department of Pharmaceutical Sciences, University of Pavia, 27100 Pavia, Italy
[*] Carmen Lammi, Department of Pharmaceutical Sciences, University of Milan, via Mangiagalli 25, 20133 Milan, Italy. Tel +39025031992, carmen.lammi@unimi.it

Materials and Methods

Optimization of the protein extraction protocol from C. pyrenoidosa

Protein extraction was optimized by varying both solvents and physical extraction methods. In detail, two solvents were used, i.e 8 M of Urea lysis buffer and 100 mM of Tris-HCl buffer. For both of them a ratio of 1:20 (biomass/solvent) was used. The extraction was conducted using the following methods: i. stirring for two hours, ii. Ultrasonication, iii. Ultrasonication + heating according to the Scheme A. The results indicated the higher efficiency in protein extraction provided by the ultrasonication plus heating method (Scheme B).

A)

Samples	Physical Method	Lysis Buffer	Concentration (mg/mL)
C1	Stirring for 2h	8 M of Urea	11.91
C2	Ultrasonication		15.94
C3	Ultrasonication + heating		18.34

C4	Stirring for 2h	100 mM Tris-HCl buffer	0.75
C5	Ultrasonication		3.90
C6	Ultrasonication + heating		1.42

Scheme B)

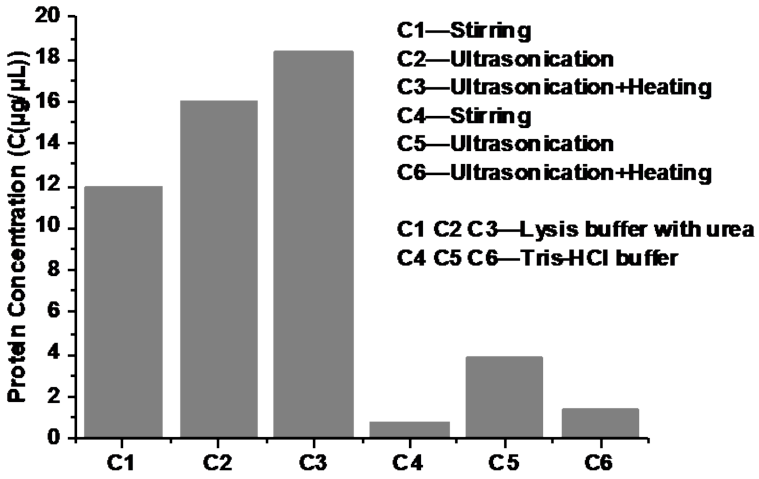

Cell cultures

Caco-2 cells, obtained from INSERM (Paris, France), were routinely sub-cultured at 50% density and maintained at 37 °C in a 90% air/10% CO2 atmosphere in DMEM con-taining 25 mM of glucose, 3.7 g/L of NaHCO3, 4 mM of stable L-glutamine, 1% nones-sential amino acids, 100 U/L of penicillin, and 100 µg/L of streptomycin (complete me-dium), supplemented with 10% heat-inactivated fetal bovine serum.

In vitro measurement of the ACE inhibitory activity

Both peptic and tryptic hydrolysates were tested for determining their ACE-inhibitory activity. For each sample, 100 µL of 2.5 mM hippuryl-histidylleucine (HHL) in buffer 1 (100 mM Tris-HCOOH, 300 mM NaCl pH 8.3) was mixed with 30 µL of sample in buffer 1 at different concentrations. Samples were preincubated at 37 °C for 15 min, then 15 µL of ACE solution, corresponding to 3 mU of enzyme in buffer 2 (100 mM Tris-HCOOH, 300 nM NaCl, 10 µM ZnCl2, pH 8.3), were added. This reaction system was incubated for 60 min at 37 °C and then stopped with 125 µL of 0.1 M HCl. The aqueous solution was extracted twice with 600 µL of ethyl acetate. The organic phase was collected and evaporated at 95 °C. The residue was dissolved in 500 µL of buffer 1 and then analyzed by a HPLC 1200 series, equipped with an autosampler (Agilent Technologies, Santa Clara, US). A Lichrospher 100, C18 column (4.6 mm × 250 mm, 5 µm; Grace, Italy) was used with water and acetonitrile as solvent following the gradient: 0 min 5% acetonitrile, 10 min 60% acetonitrile, 12 min 60% acetonitrile, 15 min 5% acetonitrile. Injection volume was 10 µL, wavelength 228 nm, flow 0.5 mL/min. The evaluation of ACE inhibition was based on the comparison between the concentrations of HA (hippuric acid) in the presence or absence of an estimated inhibitor. The phenomenon of autolysis of HHL to give HA was evaluated by a reaction blank, i.e., a sample with the higher evaluated inhibitor concentration and without the enzyme. The percentage of ACE inhibition was computed considering the area of HA peak with the following formula:

$$\%\ \text{ACE inhibition} = \left[\frac{(A_{IB} - A_{N})}{(A_{IB} - A_{RB})}\right] \times 100$$

where A_{IB} is the area of HA in inhibitor blank (IB) sample (i.e., sample with enzyme but without any estimated inhibitor), A_{N} is the area of HA in the n samples containing different amounts of the estimated inhibitor (in our case the hydrolysate), and A_{RB} is the area of HA in the reaction blank (RB) sample (i.e., sample without enzyme and with the estimated inhibitor at the highest concentration).

Cellular measurement of the ACE inhibitory activity

ACE1 Activity Assay Kit (Biovision, Milpitas Blvd., Milpitias, CA, USA) was used to evaluate the ACE inhibitory activity. Briefly, the cells were scraped in 30 μL of ice-cold ACE1 lysis buffer and transferred in an ice-cold microcentrifuge tube. After centrifugation at 13,300 *g* for 15 min at 4 °C, the supernatant was transferred into a new ice-cold tube. Total proteins were quantified by Bradford method, and 2 μL of the supernatant (the equivalent of 2 μg of total proteins) were added to 18 μL of ACE1 lysis buffer in each well in a black 96-well plate with clear bottom. For the background control, 20 μL of ACE1 lysis buffer were added to 20 μL of ACE1 assay buffer. Then, 20 μL of diluted ACE1 substrate [o-aminobenzoyl peptide (Abz based peptide) substrate, 4% of ACE1 substrate in assay buffer] was added in each well except the background one. The active ACE, cleaving the Abz-peptide (substrate), led to the release of the fluorophore and fluorescence signal (ex./em. 330/430 nm) was measured in a kinetic mode for 10 min at 37 °C.

***In vitro* measurement of the DPP-IV inhibitory activity**

The *in vitro* experiments were carried out in a half volume 96 well solid plate (white) using conditions previously optimized [1]. Each reaction system (50 μL) was prepared containing 1 × assay buffer [20 mM Tris-HCl, pH 8.0, containing 100 mM NaCl, and 1 mM EDTA]; CP or CT hydrolysates at the final concentration of 1.0, 2.5, and 5.0 mg mL^{-1}), sitagliptin at 1.0 μM (positive control), or vehicle (C, H_2O); and purified human recombinant DPP-IV enzyme. Subsequently, the mixed reagents were transferred in each well of the plate and the reaction was started by adding 50 μL of substrate solution (5 mM H-Gly-Pro-7-amido-4-methylcoumarin (AMC)) to each well and incubated at 37 °C for 30 min. Fluorescence signals were collected using the Synergy H1 fluorescent plate reader from Biotek (ex./em. wavelengths 360/465 nm).

MTT assay

A total of 3 x 10^4 Caco-2 cells/well were seeded in 96-well plates and treated with 0.1 – 5.0 mg/ml of CP and CT, or vehicle (H_2O) in complete growth media for 48 h at 37 °C under 5% CO_2 atmosphere. Subsequently, the treatment solvent was aspirated and 100 µL/well of 3-(4,5-dimethylthiazol-2-yl)-2,5-diphenyltetrazolium bromide (MTT) filtered solution added. After 2 h of incubation at 37 °C under 5% CO_2 atmosphere, 0.5 mg/mL solution was aspirated and 100 µL/well of the lysis buffer (8 mM HCl + 0.5% NP-40 in DMSO) added. After 10 min of slow shaking, the absorbance at 575 nm was read on the Synergy H1 fluorescence plate reader (Biotek, Bad Friedrichshall, Germany).

Statistical analysis

All liquid chromatography−mass spectrometry (LC−MS) analyses were run in trip-licate. For the experiments aimed at evaluating the bioactivities, all measurements were performed in triplicate and results were expressed as the mean ± standard deviation, where p-values < 0.05 were significant. Statistical analyses were performed by t-student of Mann-Whitney and one-way ANOVA (GraphPad Prism 9, GraphPad Software, La Jolla, CA, USA) followed by Dunnett's and/or Tukey's post-hoc test.

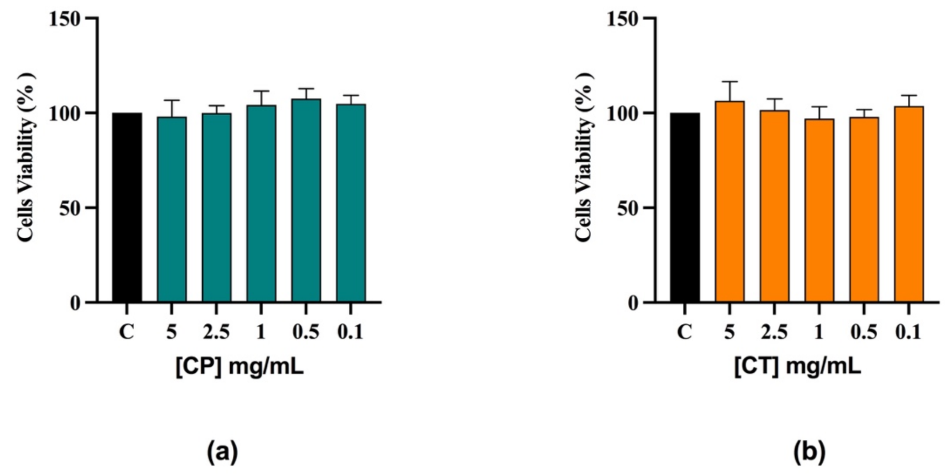

Figure S1. Effects of CP (a) and CT (b) on HepG2 cell viability assessed by MTT experiments.

Results

Table 1S. LC-ESI-MS/MS based identification of tryptic (A) and peptic (B) peptides from *C. pyrenoidosa* protein hydrolysis.

(A)

No	Protein name	Accession no	m/z (charge)	Start	Peptide sequence	Spectrum Intensity	% AA Coverage
1	Leucine-tRNA ligase	A0A087SIG7	540.19	172	(Q)YAIQTGTHPAATTATN(I)	2.84E+07	1.2
2	CTP synthase	A0A1D1ZND9	646.42	386	(K)IAAAQYARTHGVPYFGIC(L)	3.93E+07	3
3	Expressed protein	E1ZPI4	672.68	88	(M)WALGARHLVDHNATELVN(L)	3.14E+07	3.9
4	Uncharacterized protein	E1ZHY9	891.37	709	(L)LGALRGDEDGGGGGGGGSWRGRGRKGTE(-)	1.20E+07	3.8
5	Uncharacterized protein	E1ZF52	967.68	375	(F)YAPCLANRPKGDEPPQASGPGLESFPDS(L)	1.20E+07	5.9

6	Uncharacterized protein	A0A087SUF8	473.63	1	(-)MSANHDAGGS(Y)	1.24E+07	3.8
7	Uncharacterized protein (Fragment)	E1ZS03	563.12	916	(E)LMQQLEAAAAVVGEKE(L)	1.08E+07	1.6
8	Carboxypeptidase	E1ZNB5	647.06	2350	(C)YLNLPEVQEALGVAPGLR(F)	2.97E+07	3.2
9	Uncharacterized protein	M1HUP1	887.93	5	(T)FAPRFNDGRSTAREDFHEPLPVC(L)	1.39E+07	5.9
10	Glucose-methanol-choline oxidoreductase	A0A248QE08	658.71	264	(T)FQVMQDKGTRADMYRQ(Y)	3.01E+07	2.4
11	Serine/threonine-protein kinase	M1HU78	473.99	5	(T)FLKPLGSGK(Y)	2.75E+07	2.8
12	Uncharacterized protein (Fragment)	A0A1D2A4B0	661.47	1148	(D)LGGRCAGEAGALCPPRDLLN(L)	6.71E+07	1.3
13	Uncharacterized protein	A0A087SFH1	690.05	12	(T)LCRLPRGEHHPVSKPTQL(F)	3.14E+07	13.3
14	Uncharacterized protein	E1ZHZ3	615.36	18	(R)LTDRRIDGSLAAAKGTAE(L)	1.45E+07	4.4
15	TGACG-sequence-specific DNA-binding protein TGA-1B	A0A087SLT2	960.21	35	(H)LVVLEDVRASPSASAVQFPLTHQALDD(L)	1.54E+07	5.5
16	Uncharacterized protein	A0A1D1ZZN7	686.95	213	(H)LGRLLVAGAYPVAIAWQME(Y)	2.19E+07	6
17	Uncharacterized protein	A0A1D2A7Q0	922.44	81	(F)LCVAAATVPGGWAFTHPDECTCGGISAM(L)	7.44E+07	7
18	ABC transporter A family member 2	A0A087SS55	702.68	680	(H)LHVFAAIKGIPAATRAAEAAK(L)	1.22E+07	2
19	Putative sugar phosphate/phosphate translocator	A0A087S9M6	837.29	114	(T)YENPALPLPTPPLTSLPPPNPFH(L)	6.77E+07	10
20	Cation/calcium exchanger 4	A0A087SNT9	856.45	263	(L)LDPRVMHAPPLAAFEVRGRYRD(L)	2.31E+07	3.8
21	Cytosine-specific methyltransferase	M1HEI8	989.07	229	(E)WKPNGYSVNGVISTFHIKHPTRSPEN(I)	1.36E+07	7.5
22	Uncharacterized protein	E1ZTT2	948.67	695	(L)LGWSLSDGVAQTEAPQGGASHMVRFAGN(L)	8.37E+06	3.5
23	Uncharacterized protein	M1HE87	280.94	254	(Q)LLTKS(I)	2.72E+07	1.7
24	Uncharacterized protein	E1ZIX6	785.38	61	(I)LDQDVSGFAAAAAAAAERGPVAAPQ(L)	2.81E+07	4.5
25	Uncharacterized protein	E1ZIV7	841.11	45	(F)ILSVMGVLDTGKGLIREGSGSAVFN(L)	1.34E+07	14.3
26	Glycosyl transferase	M1IGC6	900.21	84	(Y)IDSAAVFERPITPYAEHVTDEKPI(L)	2.10E+07	2.8
27	Broad-range acid phosphatase DET1	A0A087SH24	281.04	445	(R)LLGRC(L)	5.09E+08	0.6
28	Uncharacterized protein	A0A1D2ABY3	864.85	595	(Y)LDRGKTVIVYCDTRESPEACAHE(I)	1.70E+07	2.1
29	LAGLIDADG homing endonuclease	A0A1Z1GBL4	578.56	125	(K)WKKHSKAIRFCTDN(F)	1.06E+07	6.8
30	Uncharacterized protein C9orf78	A0A087SE57	541.66	13	(T)LDEAHESGEEDAPPK(L)	1.92E+07	5.9
31	Uncharacterized protein PDC1	E1ZFT1	926.51	113	(R)LKMISCCNELNAGYAADGYGRANGIAC(L)	1.84E+07	4.2

32	Hypoxanthine phosphoribosyltransferase	E1ZD74	887.38	77	(S)YGAGTVSSGKVALTMVGGTDVKGRHVL(L)	1.58E+07	14.4
33	GIY-YIG catalytic domain-containing endonuclease	M1HCV1	281.64	488	(C)LLSKT(W)	5.31E+08	1.5
34	Type I inositol 1,4,5-trisphosphate 5-phosphatase 12	A0A087SPV7	917.24	117	(M)LRLIALAQSEHPFTSKNADVALSPAT(W)	2.48E+07	2.1
35	Glucosamine--fructose-6-phosphate aminotransferase	M1I2B0	961.26	174	(K)YKSPLVIGMNADGSICIASDPIATTTDK(I)	3.48E+07	4.7
36	Uncharacterized protein	E1ZL33	591.15	290	(I)WDTQPPQPTITTSGGGK(F)	6.82E+06	0.7
37	Uncharacterized protein	E1ZLR1	805.23	7	(S)WRPCKSDTVVERRVATAAPRD(W)	3.21E+07	2.7
38	Uncharacterized protein	E1ZND2	857.67	462	(L)LEVFARMYNQRPAVDYAVVAQM(L)	9.82E+06	2.8
39	Uncharacterized protein (Fragment)	A0A1D1ZUE1	941.25	36	(S)WGTCARCSGPLTGALWTSGLVCVLLSGI(L)	1.56E+07	7.2
40	Uncharacterized protein	E1Z5N4	846.75	882	(E)LWRDPNCAGSLTAAAQAAPDTPAGAL(F)	3.52E+07	1.8
41	Palmitoyl-monogalactosyldiacylglycerol delta-7 desaturase, chloroplastic	A0A087SFK9	991.24	499	(W)FVNSAAHVWGSQSYRTGD(L)	8.78E+06	3
42	Uncharacterized protein	E1ZHA1	843.2	216	(L)LNDQSTILPSGAKEPD(Y)	4.68E+07	2.5
43	Uncharacterized protein	E1ZK58	883.58	644	(S)LSCFADGTPVFALADADGAFASTVFR(L)	1.80E+07	2.7
44	Uncharacterized protein	E1ZIM1	867.57	732	(Q)LLPLADQHLRVEATGAAQQARVLM(L)	1.18E+07	2
45	Enhancer of polycomb-like protein	E1Z3I7	814.44	1	(-)MSRAFRARPLDVSRPLELIVD(L)	2.26E+07	2.8
46	Uncharacterized protein	E1Z511	938.57	752	(M)LNHGPAAAANAAFDMCPLPAALPPSLQR(Y)	1.80E+07	2.8
47	Uncharacterized protein	E1ZMI6	941.39	134	(Y)FEKRQPFNPIAFLKTPYGMMGAFM(L)	1.99E+07	11.7

(B)

No	Protein name	Accession no	m/z (charge)	Start	Peptide sequence	Spectrum Intensity	% AA Coverage

1	ATP synthase subunit beta	F2YGR0	747.35	256	(K)QDVLLFIDNIFR(F)	1.92E+08	2.4
2	ATP synthase subunit beta	A0A087SBN0	955.5	259	(R)DEEGQDVLLFVDNIFR(F)	1.19E+08	1.1
3	Elongation factor 1-alpha	A0A087SK74	622.45	42	(R)LLFELGGIPER(E)	1.41E+07	2.3
4	Acyl carrier protein	E1Z5W8	595.97	75	(K)ISTVQEAADLIAAQIDK(-)	2.21E+07	18.6
5	Elongation factor Tu, chloroplastic	P56292	723.83	216	(R)ETEKPFLMAVEDVFSITGR(G)	1.40E+08	4.6
6	Photosystem II CP47 reaction center protein	A0A2I4S6M8	929.56	311	(R)YQWDLGFFQQEIER(R)	2.62E+07	2.5
7	Uncharacterized protein	E1Z395	874.89	19	(K)AAGIEVEPYWPGLFAK(L)	2.74E+07	17.3
8	Chlorophyll a-b binding protein, chloroplastic	E1ZLI4	820.73	59	(K)GEFPGDYGWDTAGLSADPETFAR(Y)	1.63E+07	8.9
9	3-ketoacyl-CoA thiolase 2, peroxisomal	A0A087SPH9	685.8	106	(K)DTPVDDLIAAVLK(D)	3.52E+07	2.8
10	Ribulose bisphosphate carboxylase large chain	F2YGL1	601.28	218	(R)FLFVAEAIYK(S)	2.78E+07	2.1
11	Expressed protein	E1ZAF0	713.34	333	(K)LDAAPEDDHITLMRNGTLR(L)	5.65E+07	2.6
12	Uncharacterized protein	E1Z7I0	917.42	707	(R)ATAAAGDVDSVLAGIDRFAGYYPMYR(S)	4.50E+07	2.6
13	Uncharacterized protein	E1ZJA2	737.01	31	(R)GITFGRAGFTTGTGDGLVVVQR(Y)	7.15E+07	7.8
14	DNA excision repair protein ERCC-6-like protein	A0A087SA08	916.63	311	(R)LLAGGYSFEIQEHLAHASFIPGFSR(A)	4.54E+07	1.6
15	WD repeat-containing protein 74	A0A087SLF6	697.1	131	(R)QPDDGAWAEAWRWQAACK(E)	5.92E+07	5.6
16	Uncharacterized protein	E1ZA50	916.79	71	(K)SGGCHSDHSTPGSSGSAAAHGAAHGPPPPR(Q)	5.55E+07	4.3
17	Expressed protein	E1Z3R5	903.94	229	(R)QELRCQGSCHSDTIDCMRFLPGGR(L)	1.85E+07	5.8
18	Putative histone-lysine N-methyltransferase ATXR3	A0A087SSG3	686.73	342	(R)DAWGMDCYTRR(N)	3.82E+07	0.8
19	Uncharacterized protein	E1Z1Z7	891.36	134	(R)DYFLPIVADAEAGFGGPLNAYELTK(H)	6.00E+07	5.9
20	Putative polygalacturonase	A0A087SUF0	700.16	139	(R)ALVDWADPSCPVPSECRPR(L)	3.77E+07	4.3
21	Uncharacterized protein	E1Z9B3	700.16	12	(R)GEEDLLKIQQLLDEALDR(R)	3.85E+07	5.2
22	Uncharacterized protein (Fragment)	E1ZMP5	809.48	272	(K)GLLNLIDLAGSERLSRSAVTGER(L)	7.36E+06	6
23	DNA polymerase epsilon catalytic subunit A	A0A087SA98	745.04	1923	(R)LLQTHGEKLEVSAEELNAPR(F)	2.76E+07	0.9

24	Uncharacterized protein	E1ZBW6	773.42	633	(R)GPFKGGGGGGGGSGARGSGVEAAGQER(D)	9.52E+06	4
25	Thymidylate synthase	M1IKB3	801.03	162	(K)ESARMVLPMSSPTTIYMTGTAR(S)	9.81E+06	9.7
26	Uncharacterized protein	E1ZQZ4	617.97	22	(R)LEQAQSEHSQLKEAVR(S)	3.87E+07	4.6
27	Biotin carboxyl carrier protein of acetyl-CoA carboxylase	A0A087SAZ6	698.86	189	(R)VGKGPLAAAGDSVKK(G)	6.59E+07	6.3
28	Uncharacterized protein	A0A1D1ZW70	840.83	383	(R)LYRVDIQLAFPEASTKALTAGVR(Q)	1.24E+07	3
29	Uncharacterized protein (Fragment)	A0A1D1ZQ66	855.86	160	(R)LGGHGGARARHHAAHHAGGDADALPR(G)	4.04E+07	6.3
30	PBCV-specific basic adaptor domain-containing protein	M1HTF0	972.53	205	(K)INIQGTNTGAFKKVYSNFIFPIQTSK(G)	1.28E+07	5.2
31	Uncharacterized protein Z225R	A7K8I5	637.27	146	(K)DTIQRFAREQLNDFR(Y)	3.86E+07	4.3
32	Uncharacterized protein	E1ZTL8	654.31	59	(R)IVAGYQLNLATEEVERR(L)	9.71E+06	7.7
33	Pre-mRNA-splicing factor SYF1	A0A087SH90	926.15	394	(K)QILCYTEAVRTVDPDK(A)	1.18E+08	1.7
34	Uncharacterized protein b105L	A7IVY0	942.22	75	(R)QASRLIGIAREAILFSIDNLSSHGTR(N)	2.64E+07	11.3
35	Prostaglandin E synthase 2	A0A087SK04	923.61	131	(K)TPLPLPESIVLYQYEVCPFCCKVK(A)	6.66E+07	8.4
36	Uncharacterized protein	E1ZP51	281.11	175	(R)LLSTK(A)	5.17E+08	2.1
37	Uncharacterized protein	E1Z860	713.91	208	(R)VMPLREGGADIPVTEENRR(E)	6.35E+07	4.8
38	Uncharacterized protein	M1HZD6	775.8	236	(K)ILAETDDVSADVAPAIQIILEK(T)	4.98E+06	7.3
39	Uncharacterized protein	E1ZDE7	922.88	207	(K)IEALRAIMERPVAANGATLHFVDDR(Y)	4.00E+07	8
40	Uncharacterized protein M629L	A7IV09	831.93	199	(R)EHNTAGGFLDHLVSSIAGEVERR(L)	6.09E+06	9.9
41	Elongation factor G, mitochondrial	E1Z1Z5	781.03	362	(R)VLLPCSKTGPLVALAFKLEEGR(F)	4.88E+07	2.8
42	Uncharacterized protein	M1HK68	812.2	190	(K)RAFSLVRPFDNSLVTTEIVDR(N)	3.57E+07	8
43	Putative serine/threonine-protein kinase GCN2	A0A087SFY1	576.18	105	(R)DVAHEMALKREAEAR(R)	1.67E+07	1.1
44	Uncharacterized protein (Fragment)	A0A1D1ZYR9	280.96	1	(-)QIYTMGK(D)	7.32E+08	1.2
45	Uncharacterized protein	E1Z459	792.23	1755	(R)QAADGPAGGGGVVVDLVGGGAAPVRVK(R)	1.10E+08	1.4
46	Abnormal spindle-like microcephaly-associated protein-like protein	A0A087SMF7	836.46	252	(R)LVDALTGRPGLALQTEIDRLAAGR(G)	1.90E+07	2
47	Protein arginine N-methyltransferase	A0A087SR29	967.19	371	(R)MYAVEKNPNAIVTLSHRVETEWQGK(V)	2.11E+07	4
48	Uncharacterized protein	E1Z6X6	630.59	250	(R)NREALYAVSGALERAAAK(R)	1.35E+07	3.4

49	Uncharacterized protein	A0A1D2A538	838.43	1	(-)MALGPTVIRGNASEIMALASLGGER(T)	5.89E+07	11.7
50	Uncharacterized protein	A0A1D2ACR1	990.94	32	(R)SITVPRPISAPTYAPEDPKGRSFVQTR(Y)	6.68E+06	6.6
51	Gene, similar to reverse transcriptase genes of various retrotransposons	O64463	713.62	56	(R)LIDAAAEFCEQTGMVISVDK(T)	8.21E+07	10.4
52	Transmembrane protein 56-A	A0A087SMF8	923.57	260	(R)TLKPEELDSSIGAAFGAPRHDVSKDK(D)	6.83E+06	13.4
53	Uncharacterized protein	E1ZE84	926.51	712	(K)IPVSPEEHQPALPPATLRPETAAEAR(A)	3.59E+07	2.8
54	Uncharacterized protein	E1ZJF1	906.17	201	(K)LRAEVAAAEGAWLPPWAAASLGPALAR(T)	2.14E+07	6.3
55	Uncharacterized protein	E1ZCL5	474.11	472	(R)EAERGGDGR(G)	2.00E+07	1.6
56	Uncharacterized protein	A0A1D2AD70	684.99	327	(R)KLIIGSLPVSLFCSGQTYK(E)	1.43E+07	2.9
57	Uncharacterized protein	E1ZFA8	280.96	267	(R)ILGCR(F)	2.01E+09	0.4
58	Chaperone protein dnaJ 10	A0A087SRC2	988.59	197	(R)FQRLGQAYQVLGNPDLR(A)	1.49E+07	4
59	Uncharacterized protein	A0A1D2A1J8	941.35	229	(R)LLQTAPQLLPAAVEAFYYRDLDDAK(A)	1.40E+07	4
60	Hydrogenase	E7BYE2	617.29	200	(K)QGEADREWFNTTGLAR(D)	2.72E+07	3.6
61	Protein DGCR14	A0A087SQK6	871.89	178	(K)SAAGPRPTDGFGTGGQPSDRLVQWR(Y)	3.81E+07	7.2
62	Uncharacterized protein	E1ZPJ5	663.54	110	(K)QQEEGERLRAEGDGGGDGK(G)	1.22E+07	5.2
63	Peptidyl-prolyl cis-trans isomerase, chloroplastic	A0A087SB74	955.91	46	(R)YALPIHNTPIRQVQESLESISEALR(I)	7.00E+07	2.5
64	Uncharacterized protein	E1Z9J7	886.15	39	(K)IVVVGGQSSGKSSVLEAVVGRDFLPR(G)	1.98E+07	4.2
65	Phosphatidylserine synthase 2	A0A087SSA8	280.93	420	(R)QHAGTKAK(A)	2.68E+07	1.8
66	Uncharacterized protein	A0A1D1ZZN9	706.3	889	(R)QWGGAAQREASASPAEASTSR(A)	8.03E+07	1.9

Validation and assessment of the docking protocol.

As reported in the main text, before starting docking calculations on the short peptides, the accuracy of the GLIDE docking protocol, and the parameters to apply, was established by docking the ligands co-crystallized within ACE and DPP-IV, i.e., captopril and IPI. The RMSD between the docking pose endowed with the highest score and the one found in the X-ray was calculated. Typically, RMSD values within 2 Å are considered successful, whereas values higher than 3 Å indicate docking failure [2]. For each complex (captopril-ACE and IPI-DPP-IV). **Figure S1** shows the comparison between the docking poses and crystal structures. As displayed, the docking results of captopril/ACE presents an excellent RMSD value of 0.7640 Å. The binding sites were profiled clearly in 2D interaction view (**Figures S1B** and **S1C**). In both pictures, the residues GLN281 and TYR520 were bound to the carboxyl group of captopril by hydrogen bonds, while LYS511 creates a salt bridge; His353 and His513 interact with the carbonyl group bound to the pyrrolidine ring by a hydrogen bond. Interestingly, in the docking pose, a salt bridge was generated between the zinc ion and the thiol group of captopril, as was found in the crystal structure, greatly contributing to the inhibition of enzyme activity [3,4]. Although the metal coordination was not fully predicted by docking calculations with GLIDE, the distance between the zinc ion and captopril was really close to the one found in the crystal structure (2.34 *vs* 2.37 Å).

In the case of IPI/DPP-IV complex (**Figure S1D**), the docking structure got a slightly higher RMSD of 2.7390 Å versus the crystal structure despite all the residues involved in the interaction with IPI in the X-ray complex were also involved in the docking pose of IPI. Only the conformation of the hydrophobic chains was docked differently (see **Figure S1E-F**). However, the overall orientation of IPI is largely reproduced by the docking protocol, in fact the H-bond network and the salt bridges

shaped by Glu205, Glu206 and Arg125 were found in the IPI docked pose endowed with the best glide score.

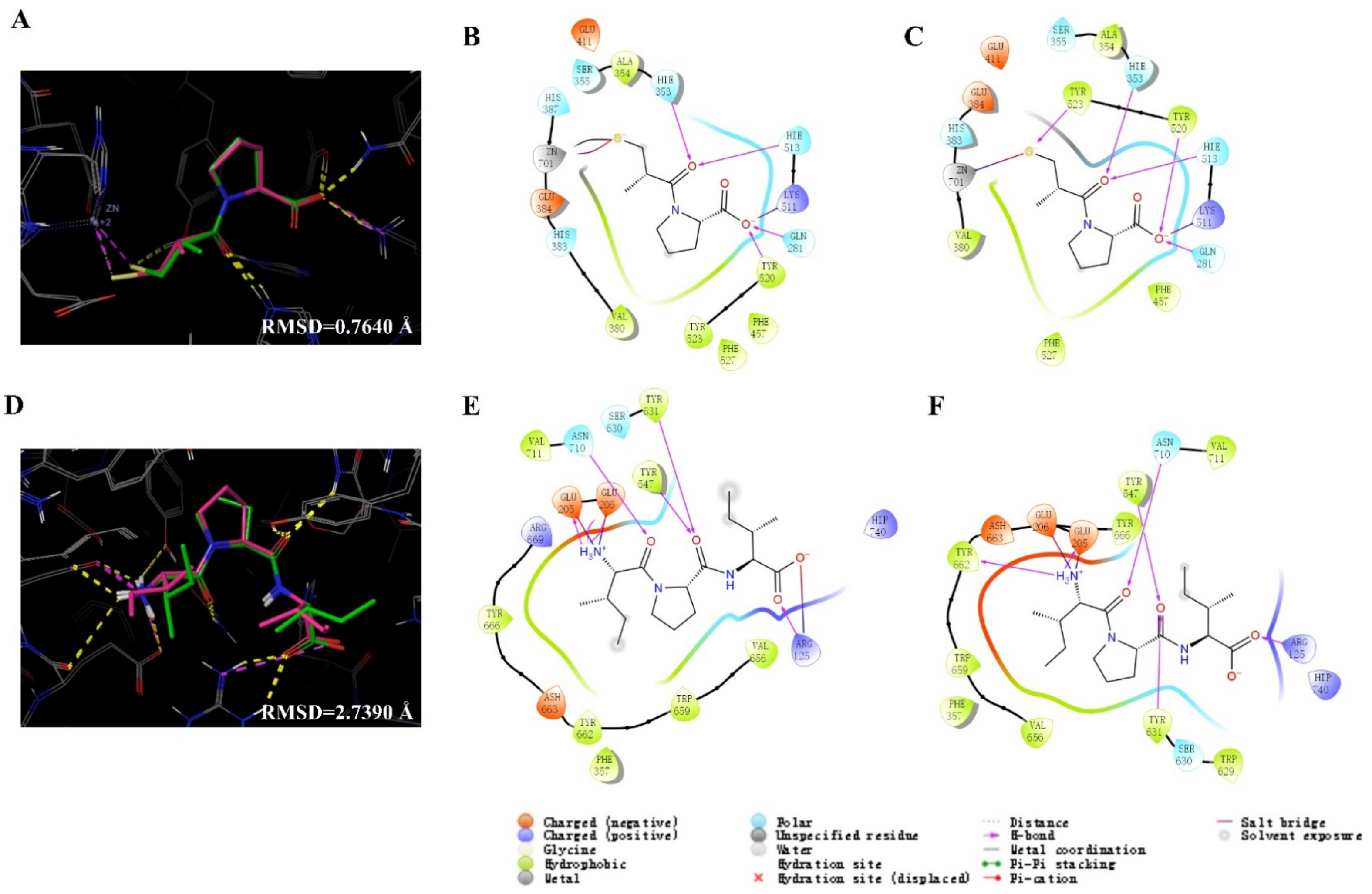

Figure S2. Validation of the docking protocol by comparing the docking pose and the crystal structures of ACE/captopril and DPP-IV/IPI complexes. For ACE: (A) the alignment of docking pose (green) and crystal structure of captopril (pink); (B) binding mode of captopril as was found in the X-ray crystal structure; (C) predicted binding mode of captopril as was found in the best docking pose. For DPP-IV: (E) alignment of docking pose (green) and the X-ray crystal structure of IPI (pink); (F) binding mode of IPI within DPP-IV as in the X-ray crystal structure; (G) predicted binding mode of IPI as it was predicted by docking calculations. In (A) and (D) figures, hydrogen bond is present as yellow line and salt bridge is purple line.

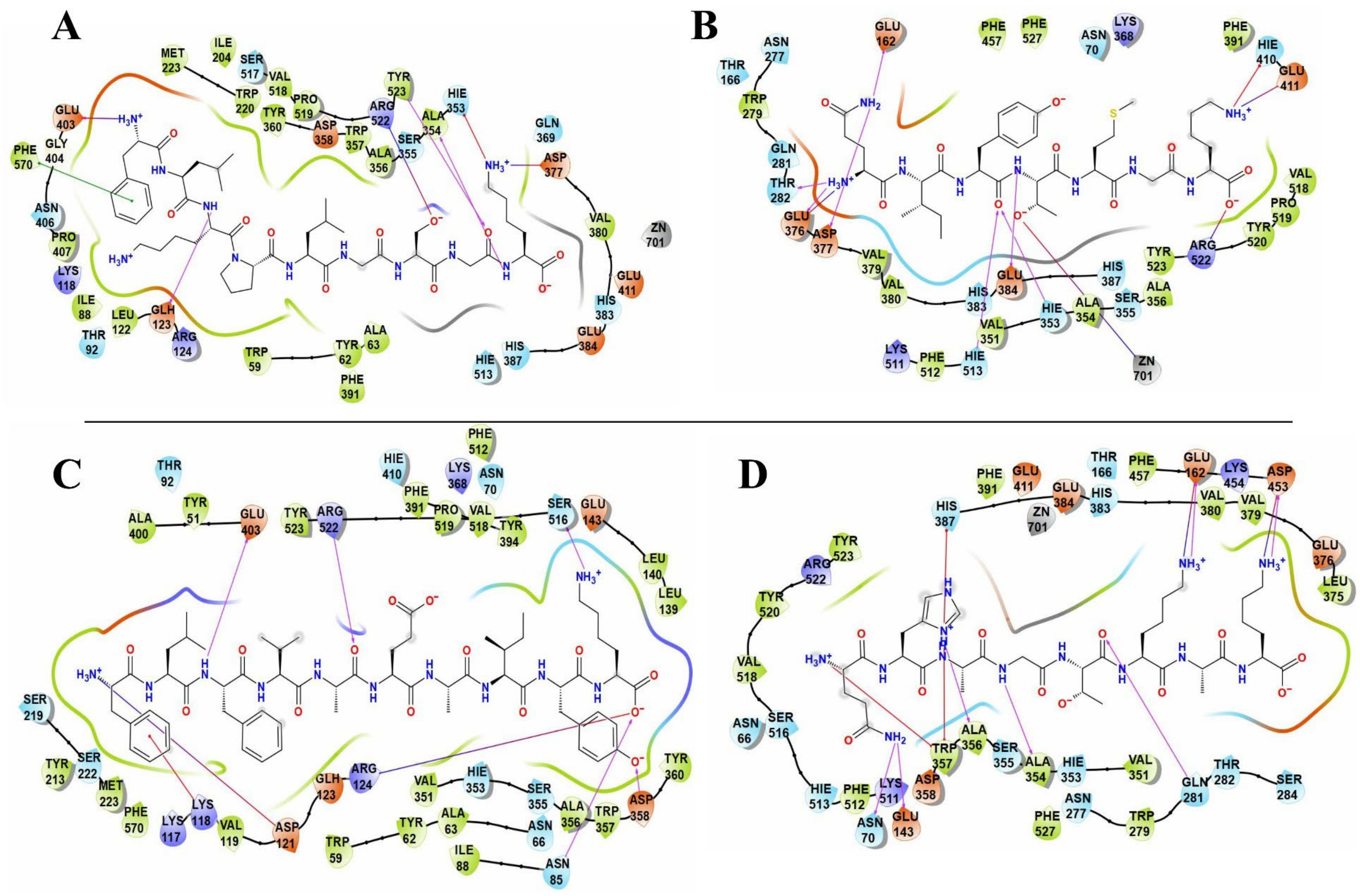

A
B
C
D
GLU 403
PHE 570
GLY 404
ASN 406
PRO 407
LYS 118
ILE 88
THR 92
LEU 122
GLH 123
ARG 124
MET 223
ILE 204
SER 517
VAL 518
TRP 220
PRO 519
TYR 360
ASP 358
TRP 357
ARG 522
TYR 523
SER 355
ALA 356
ALA 354
HIE 353
GLN 369
ASP 377
VAL 380
ZN 701
GLU 411
HIS 383
GLU 384
HIS 387
HIE 513
TRP 59
TYR 62
ALA 63
PHE 391
THR 166
ASN 277
TRP 279
GLN 281
THR 282
GLU 376
GLU 162
PHE 457
PHE 527
ASN 70
LYS 368
HIE 410
VAL 379
LYS 511
PHE 512
VAL 351
TYR 520
THR 92
ALA 400
TYR 51
HIE 410
PHE 512
ASN 70
PRO 519
TYR 394
SER 516
GLU 143
LEU 140
LEU 139
SER 219
TYR 213
SER 222
MET 223
PHE 570
LYS 117
VAL 119
ASP 121
ASN 66
ASN 85
ASP 453
LYS 454
LEU 375
ARG 522
ASN 66
THR 282
SER 284
ASN 277

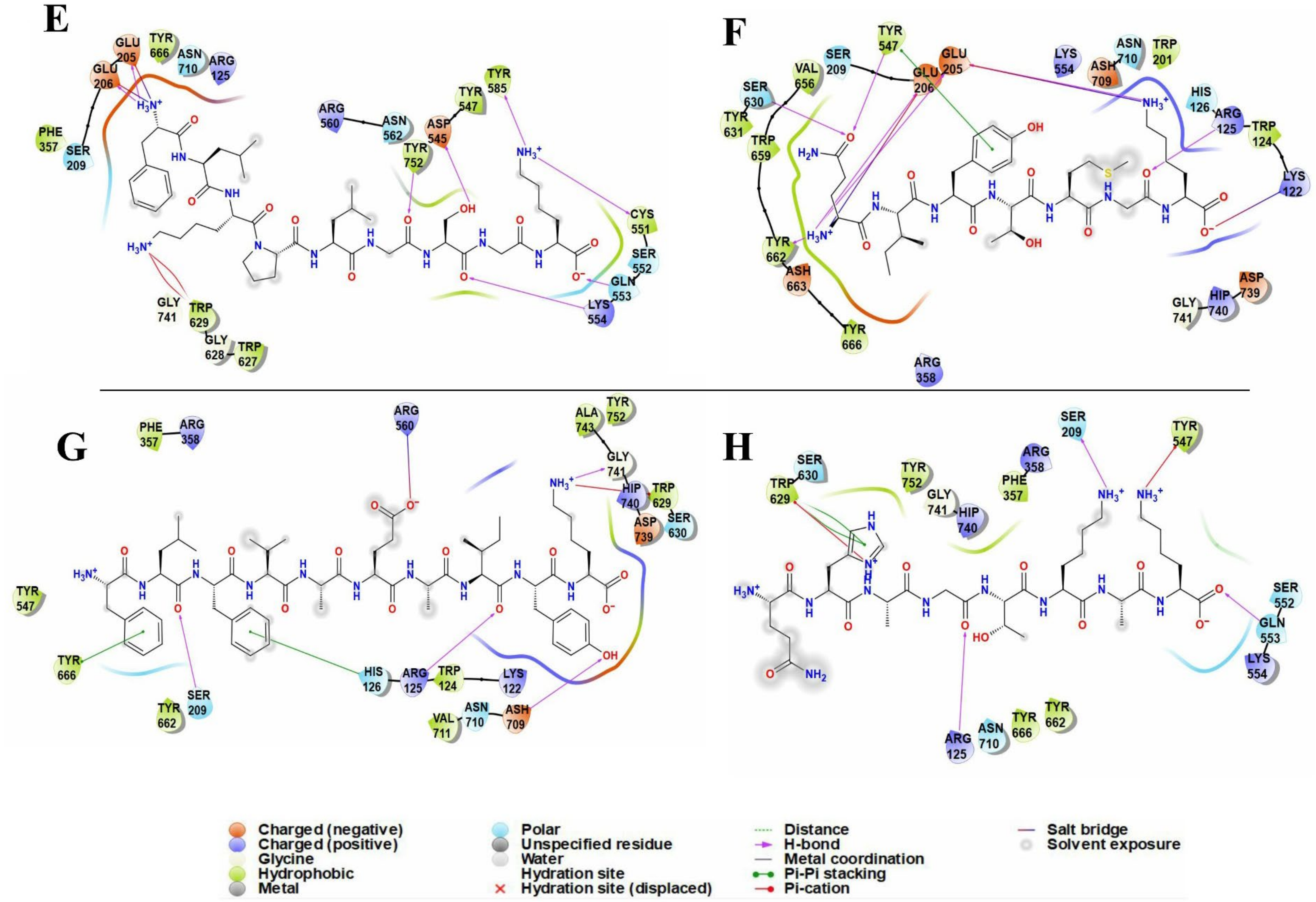

Figure S3. 2D views of the predicted binding modes between *C. pyrenoidsa* derived peptides and ACE/DPP-IV. (**A**) Pep2 (FLKPLGSGK), (B) Pep7 (QIYTMGK), (**C**) Pep8 (FLFVAEAIYK), and (**D**) Pep10 (QHAGTKAK) in complex with ACE; (**E**)Pep2 (FLKPLGSGK), (**F**) Pep7 (QIYTMGK), (**G**) Pep8 (FLFVAEAIYK), and (**H**) Pep10 (QHAGTKAK) in complex with DPP-IV.

Peptide stability evaluation of screened peptides by *in vitro* digestion. CP and CT solution (pH 2.0) were treated by pepsin (4 mg/mL in NaCl) respectively (E/S ratio 1:100) at 37 °C for 60 min with continuous shaking. Then the pH was adjusted to 8.0 with 1 M NaOH and pancreatin (4 mg/mL in ddH2O) was added (E/S ratio 1:100) to mimic the intestinal digestion at 37 °C for 15 min. The enzymatic reaction was blocked by heating at 95 °C for 10 min and the employed enzymes were totally removed by centrifugal ultrafiltration (3 kDa cutoff). Above process was repeated except the addition of pepsin and pancreatin, serving as controls. The stability of four screened peptides was assessed through multiple reaction monitoring (MRM) mass spectrometry. The injected samples were eluted as following gradients: 5% solvent B (0 min), 70% solvent B (0-10 min), and back to 5% in 5 min. The drying gas temperature was set at 300 °C, flow rate 3 L/min (nitrogen). Data acquisition was carried out in positive ionization mode. Capillary voltage was -2000 V, with endplate offset -500 V. Full scan mass spectra were acquired in the mass range from 100 to 850 Da. Capillary voltage was −2000 V, with endplate offset −500 V, skimmer -40 V, drying gas flow 5 L/min, drying gas temperature 300 °C. The targeted assay was performed by targeting the precursor ions at *m/z* 474.0 for Pep2 (FLKPLGSGK), *m/z* 601.3 for Pep8 (FLFVAEAIYK) and *m/z* 281 for both Pep7 (QIYTMGK) and Pep10(QHAGTKAK), respectively. Peptides were targeted through matching the retention time, MS profiles and MS/MS fragmentation spectra. For Pep2 and Pep8, intensity of their MS profiles was acquired to monitor the peptide stability, instead, for Pep7 and Pep10, since their m/z are quite similar, the intensity of typical fragment ions at m/z 167.9, 319.0 for Pep7 and m/z 197.4, 394.1 for Pep10 were employed

A

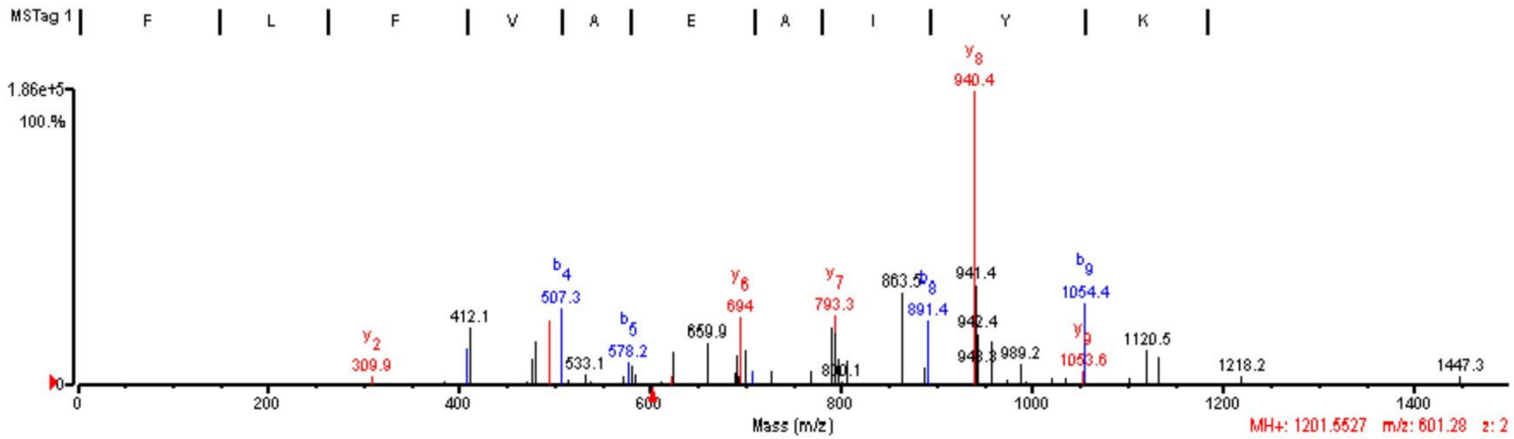
MSTag 1
F L F V A E A I Y K
1.86e+5
100.%
0
y8
940.4
b4
507.3
y6
694
y7
793.3
863.5
b8
891.4
941.4
b9
1054.4
412.1
b5
578.2
659.9
942.4
y2
309.9
533.1
989.2
y9
1053.6
1120.5
1218.2
1447.3
0 200 400 600 800 1000 1200 1400
Mass (m/z)
MH+: 1201.5527 m/z: 601.28 z: 2

B

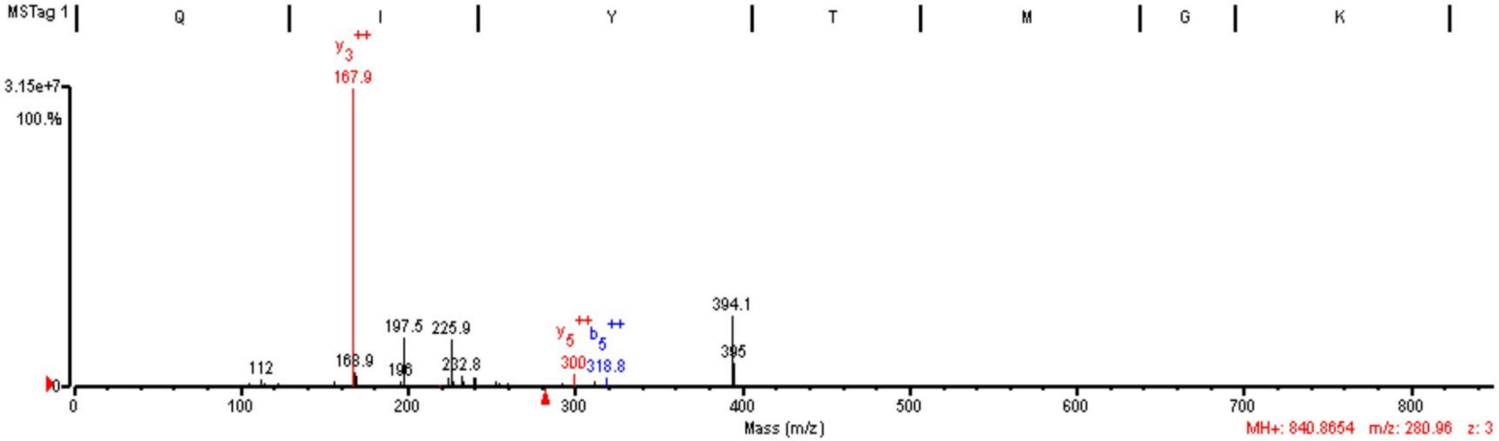
MSTag 1
Q I Y T M G K
y3 ++
167.9
3.15e+7
100.%
0
394.1
197.5
225.9
y5 ++
b5 ++
300
318.8
395
112
168.9
196
232.8
0 100 200 300 400 500 600 700 800
Mass (m/z)
MH+: 840.8654 m/z: 280.96 z: 3

C

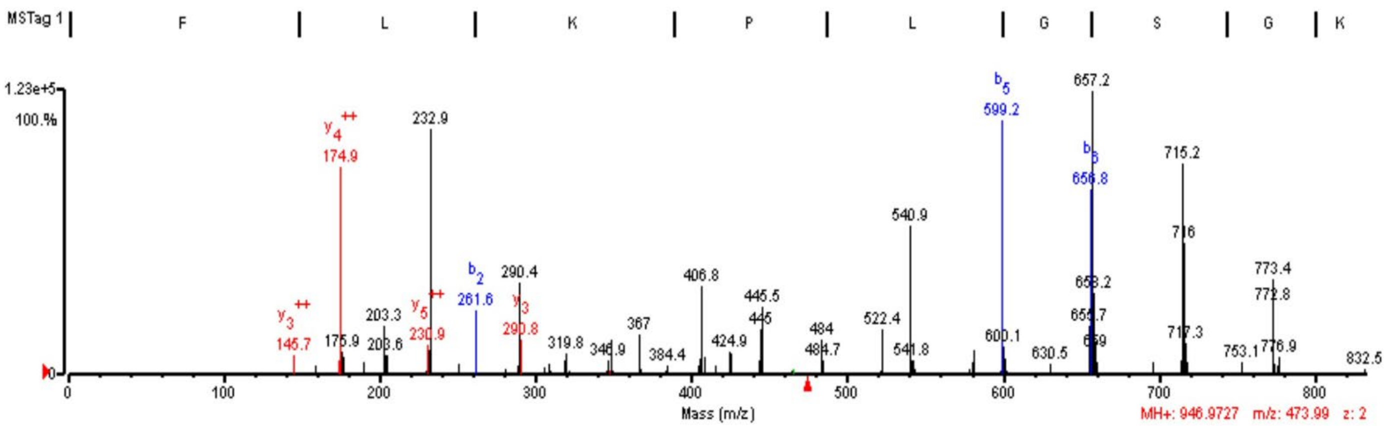

D

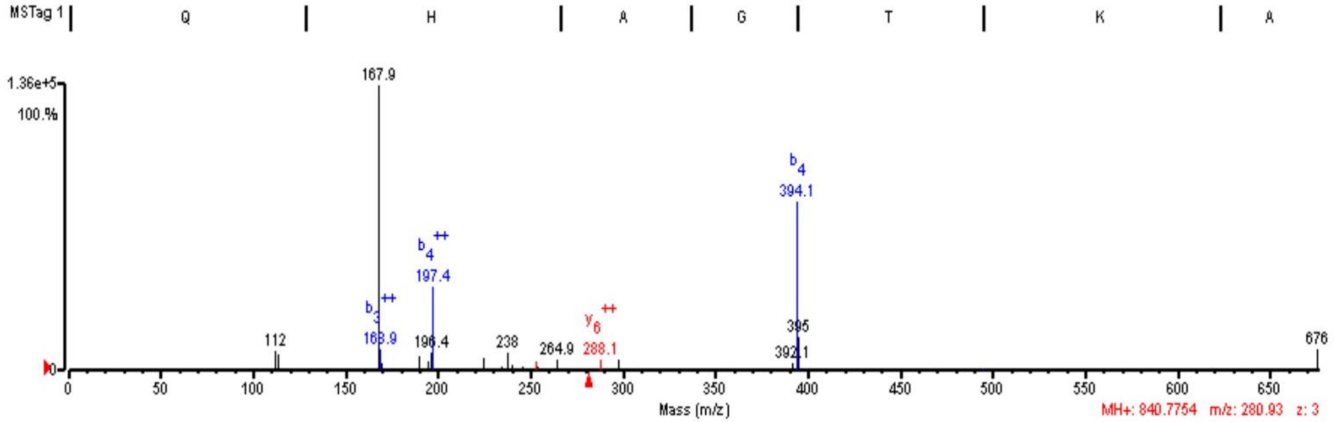

Figure S3. Electrospray ionization-tandem mass spectrometry spectra of $[M+H]^+$ ions of (A) Pep2, (B) Pep 7, (C) Pep8 and (D) Pep10, respectively.

References

1. Lammi, C.; Zanoni, C.; Arnoldi, A.; Vistoli, G., Peptides derived from soy and lupin protein as Dipeptidyl-Peptidase IV inhibitors: *In vitro* biochemical screening and *in silico* molecular modeling study. *J Agric Food Chem* **2016**, *64*, 9601-9606.
2. Ding, Y.; Fang, Y.; Moreno, J.; Ramanujam, J.; Jarrell, M.; Brylinski, M., Assessing the similarity of ligand binding conformations with the Contact Mode Score. *Computational Biology and Chemistry* **2016**, *64*, 403-413.
3. Natesh, R.; Schwager, S. L. U.; Evans, H. R.; Sturrock, E. D.; Acharya, K. R., Structural details on the binding of antihypertensive drugs captopril and enalaprilat to human testicular angiotensin I-converting enzyme. *Biochemistry* **2004**, *43*, 8718-8724.
4. Bunning, P.; Riordan, J. F., The functional-role of zinc in angiotensin converting enzyme - implications for the enzyme mechanism. *Journal of Inorganic Biochemistry* **1985**, *24*, 183-198.
